# Multimodal colour modulation for cognitive enhancement in intelligent rehabilitation: a systematic review and translational guidance

**DOI:** 10.1186/s12984-026-01991-y

**Published:** 2026-05-11

**Authors:** Lina Xu, Xingkai Wang, Wenan Li, Yuyang Jiang, Chen Cheng, Zhenhong Li, Yuyang Wang, Luwen Yu

**Affiliations:** 1 Computational Media and Arts thrust, The Hong Kong University of Science and Technology (Guangzhou), No.1 Du Xue Rd, Nansha District, Guangzhou, Guangdong China; 2 The Hong Kong University of Science and Technology, Clear Water Bay, Kowloon, Hong Kong SAR China; 3 Department of Electrical and Electronic Engineering, The University of Manchester, Manchester, M1 3BB UK

**Keywords:** Cognitive enhancement, Colour modulation, Multimodal assessment, Intelligent rehabilitation, Systematic review

## Abstract

**Background:**

Colour modulation has been investigated as a non-invasive means of supporting cognitive performance, yet translation to rehabilitation-oriented applications has been constrained by fragmented evidence, inconsistent parameterisation, and heterogeneous assessment strategies.

**Main body:**

This systematic review synthesises 75 studies published between 2011 and 2025 through a Colour-Modality-Cognition framework that links stimulus design, measurement strategy, and cognitive goals. Across the evidence base, a graded and context-dependent pattern emerged. Contrast-related variables, particularly lightness and colour difference, showed more consistent support for baseline legibility and task reachability, whereas hue- and chroma-related effects were more conditional on task family, visual context, implementation locus, and the baseline contrast conditions already in place. Across several studies, efficiency-related changes, including shorter search times or reduced cortical load, were more readily captured by ocular and neurophysiological measures than by behavioural outcomes alone, especially where behavioural performance approached ceiling levels. However, the literature rarely assessed post-training retention, transfer, or other longer-term rehabilitation-relevant outcomes.

**Conclusion:**

Current evidence supports a more explicit framework for the design and evaluation of rehabilitation-oriented colour interventions, particularly in relation to contrast-secured visual design and the use of multimodal assessment. However, implications for adaptive rehabilitation systems remain preliminary because most available evidence derives from controlled, predominantly non-clinical task settings rather than direct rehabilitation deployment.

**Supplementary Information:**

The online version contains supplementary material available at 10.1186/s12984-026-01991-y.

## Introduction

 Cognitive enhancement is a central objective in the rapidly developing field of intelligent rehabilitation. With global demographic shifts and the increasing prevalence of cognitive impairments stemming from neurological conditions such as stroke and Alzheimer’s disease, the development of intelligent, effective, and scalable rehabilitation tools has become an urgent societal need [1–3]. However, current mainstream cognitive rehabilitation methods, such as repetitive task training, typically require a high degree of active participant engagement and are often limited by high costs, low adherence, and an inability to adapt dynamically to individual needs [4–6]. These shortcomings constrain the translation of promising interventions into rehabilitation systems that are scalable, adaptive, and evaluable in practice, where durability and transfer of gains are essential.

Colour modulation has been investigated as a non-invasive and precisely controllable approach to cognitive enhancement. Previous studies suggest that colour can influence psychological and physiological responses, although these effects are often context-, task-, and display-dependent [7, 8]. Some studies have associated red with increased physiological arousal and attention-related effects, whereas blue has been linked in selected task and display contexts to calmer affective responses or more creative performance; however, such associations are not uniform and remain contingent on task family, implementation setting, and baseline visual conditions [9–11]. These findings provide a theoretical foundation for the use of colour in cognitive intervention.

**Fig. 1:**
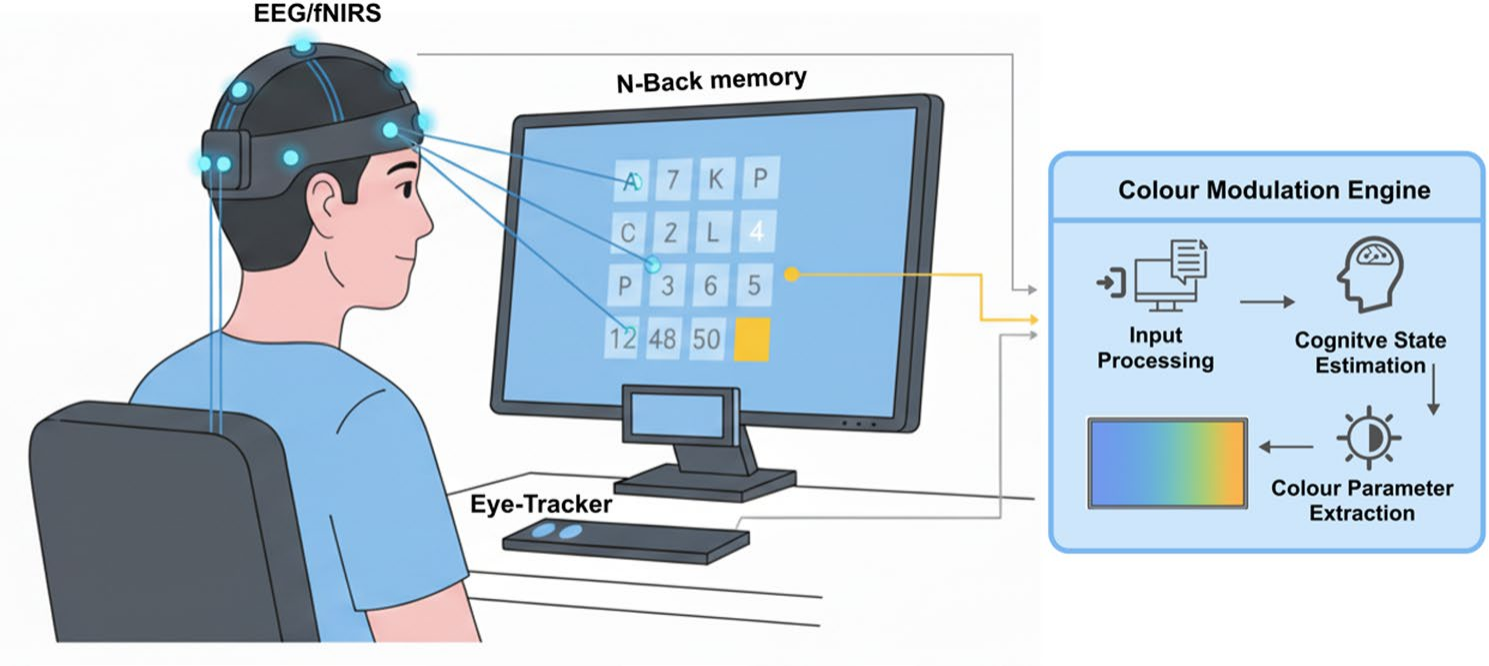
Conceptual model of a future closed-loop adaptive colour modulation system. Neurophysiological signals (e.g., EEG) from the user are processed by a ‘Colour Modulation Engine’ to analyse cognitive state. The engine then dynamically adjusts visual parameters (e.g., colour) on the display to create a real-time, adaptive interaction

Within digital environments, colour holds a unique position as a controllable lever. Unlike static aesthetic choices, colour parameters can be precisely defined, adjusted, and reproduced, enabling systematic manipulation across time and devices [12–14]. In a perceptually uniform colour space such as CIELAB, lightness (L*), hue (h°), chroma (C*), and colour difference (ΔE) offer a compact and interpretable set of variables. These variables are commonly examined in relation to core perceptual and task-related processes, for example, L* and ΔE are commonly examined in relation to legibility and task reachability [15, 16], whereas h° and C* have more often been studied in relation to affective tone, attentional orientation, or task strategy [17–19], and ΔE and C* influence attentional allocation or filtering [16, 20]. Through the dynamic adjustment of screen background hue, luminance, or foreground-background colour contrast, colour may shape cognitive load, attentional focus, and affect-related responses under some task and display conditions [15, 18, 19, 21–24]. This opens the possibility that colour could be used not only in pre-set interfaces but also in signal-informed, closed-loop interventions that adapt to users’ state. Figure 1 illustrates a conceptual model of a future closed-loop adaptive colour modulation system for rehabilitation-oriented applications. It is intended as a conceptual research framework for the subsequent synthesis and discussion.

Despite this potential, the translation of colour modulation into intelligent rehabilitation-oriented applications remains at an early stage. This constitutes a core translational gap between theory and application, stemming from the fragmented, unstandardised, and heterogeneous nature of existing research.

Specifically, the evidence base is highly dispersed, with current studies focusing on the immediate, in-task effects of colour modulation while rarely examining post-training gains, which are central to rehabilitation relevance [10, 25, 26]. This emphasis on immediate effects and the limited assessment of long-term gains represent a major translational gap in this domain [27, 28].

Furthermore, the description of colour parameters in research is often based on vague qualitative terms (e.g., red background, high saturation), rather than quantifiable values derived from a perceptually uniform colour space [10, 29–32]. This severely hinders cross-study comparison and replication.

Concurrently, assessment methods are heterogeneous and one-dimensional, with the majority of studies relying solely on behavioural metrics [20, 33–36]. As preliminary findings reveal, when performance approaches a ceiling effect, deeper changes in cognitive load or subtle shifts in attention are often overlooked. These less visible efficiency-related changes, which may be relevant to rehabilitation progress, often require multimodal assessment, including ocular and neurophysiological readouts, for more sensitive detection [21, 37–40].

To systematically address these challenges and bridge the gap between academia and application, the present review proposes an innovative *Colour–Modality–Cognition (CMC) framework*. This framework articulates three interlocking components, where *Colour* specifies the intervention tool via standardised parameters and loci of control (background manipulation, stimulus-background contrast, or dynamic shift); *Modality* defines the evaluation and feedback channels, combining task performance with physiological and ocular indicators to capture both overt outcomes and latent cognitive load; and *Cognition* identifies the targeted functions and corresponding task families (e.g., visual attention, working memory, executive control, logical reasoning, and visuospatial construction), allowing evidence to be organised by goal rather than merely by stimulus category. By structuring extraction and synthesis around these dimensions, the CMC framework supports integrative analysis and helps clarify the translational relevance of different forms of evidence.

Adhering to PRISMA 2020 guidelines, this review synthesises 75 empirical studies published between 2011 and 2025. The aim is to achieve three core objectives:

- To synthesise and evaluate the impact of colour modulation on immediate cognitive performance and the more limited evidence on longer-term outcomes (*Systematic Integration*);
- To develop a unified analytical paradigm for colour modulation research through the CMC framework, standardising key variables and providing a common model for integrating multimodal evidence (*Standardisation and Modelling*);
- To provide evidence-informed design considerations for rehabilitation-oriented research and system development, while distinguishing more direct evidence from findings that remain indirect or conditional (*Translational Guidance*).

This review consolidates a fragmented literature into a more coherent analytical framework and considers how colour may function as a component of rehabilitation-oriented design and adaptive cognitive support within the evidential boundaries of the studies retrieved and synthesised here.

## Methods

This systematic review was conducted and reported in accordance with the Preferred Reporting Items for Systematic Reviews and Meta-Analyses (PRISMA) 2020 guidelines [41]. The review used a concept-driven primary search together with a supplementary task-family-oriented validation search to improve transparency about retrieval boundaries and coverage within a predefined review scope, rather than to imply exhaustive field-wide retrieval under a single search structure.

### Primary search strategy

Web of Science, Scopus, and PubMed were searched for peer-reviewed human studies published between 2011 and July 2025. This primary search was concept-driven and covered the period during which colour manipulation and multimodal assessment became sufficiently prevalent for structured cross-study comparison. Forward and backward citation tracking of key records was additionally conducted through Google Scholar.

The primary search used three concept blocks connected by the Boolean operators AND and OR, with database-specific syntax adaptations: (1) a colour block centred on the terms colour OR color; (2) a cognition/performance block centred on cognit* OR “task performance”; and (3) translational and modality-related terms retained within the original concept-driven strategy, including multimodal, fNIRS, EEG, eye-track*, rehabilitation, and enhancement. Full database-specific search documentation is provided in the public repository as DataR6.

Inclusion required: (a) explicit manipulation of colour as an independent variable; (b) the presence of a cognitive task or task-related performance context; and (c) at least one task-relevant outcome, including behavioural, ocular, neurophysiological, subjective, or other performance-related measures. Exclusion covered non-empirical articles, non-human studies, records without a cognitive task or task-related outcome, reports without explicit in-scope colour manipulation, and studies outside the predefined review scope. Two reviewers independently screened titles and abstracts, followed by full-text assessment of potentially eligible records. Disagreements were resolved through discussion or adjudication if needed.

**Fig. 2:**
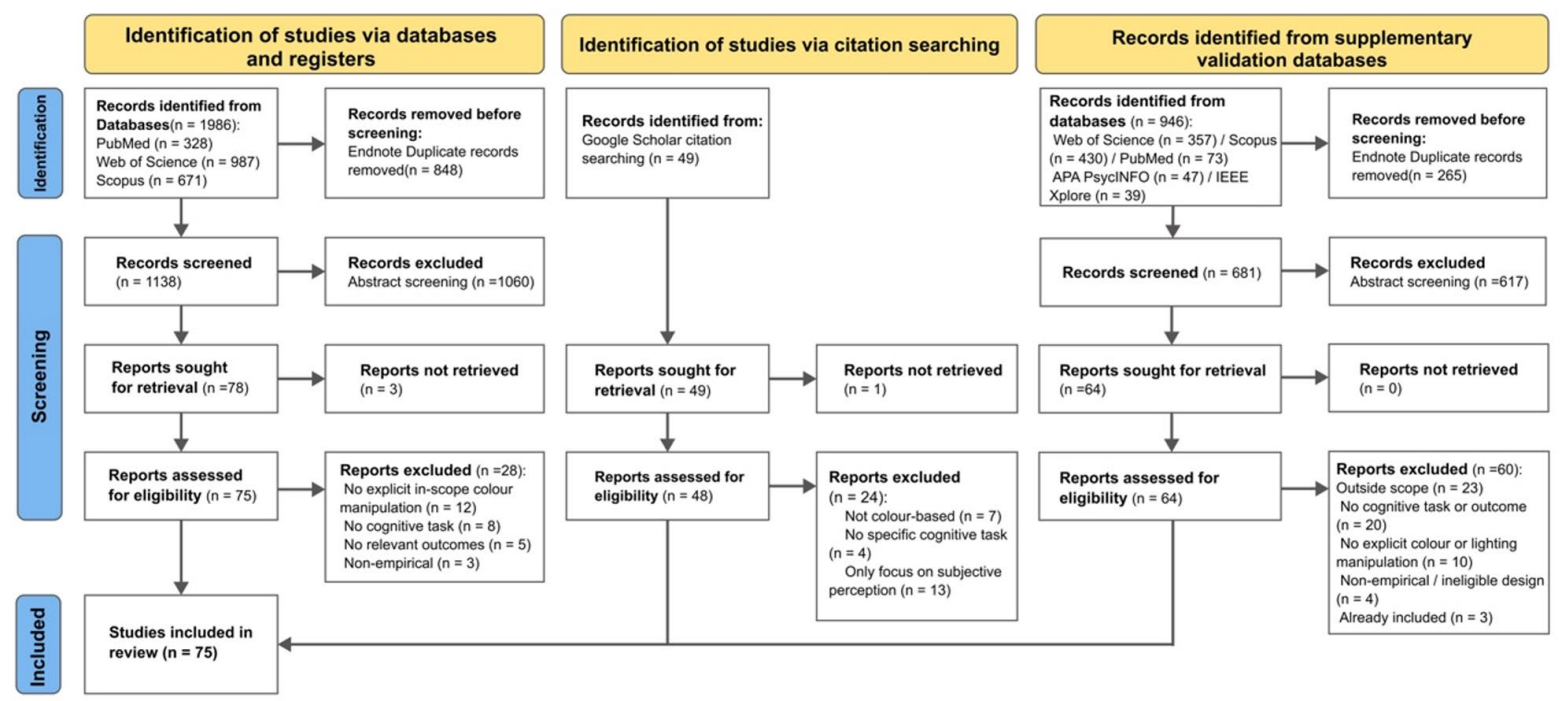
PRISMA 2020 flowchart of the article selection process

The primary search identified 1,986 database records, with a further 49 records identified through Google Scholar citation searching. After removal of 848 duplicates from the database search, 1,138 records underwent title and abstract screening, and 127 reports were sought for retrieval across database-derived and citation-derived records. Following full-text assessment, 71 studies were included in the original review set. The main reasons for full-text exclusion in the primary pathway were no explicit in-scope colour manipulation (*n* = 12), no cognitive task (*n* = 8), no relevant outcomes (*n* = 5), and non-empirical design (*n* = 3). The corresponding study-selection pathway is summarised in Fig. 2.

### Supplementary validation search and study selection

A supplementary validation search was conducted to test the coverage of the primary concept-driven search and to reduce potential retrieval bias arising from modality-specific terms. Web of Science, Scopus, PubMed, APA PsycINFO, and IEEE Xplore were searched to strengthen coverage of cognition-oriented psychological research and of interface and human-factors studies relevant to colour intervention. EMBASE was not prioritised because the review was not designed as a biomedical- or trial-focused evidence synthesis, and the primary retrieval goals were more directly aligned with multidisciplinary citation databases together with psychology- and engineering-oriented validation sources. Cochrane Library and ClinicalTrials.gov were not included because the review focused on peer-reviewed empirical studies rather than secondary syntheses or trial registrations. Grey literature was also excluded to maintain comparability of reporting standards and ensure extractable methodological detail. The supplementary validation search was designed as a coverage check on the original concept-driven strategy. It expanded task-family terms explicitly and removed any requirement for multimodal or sensor terms, while retaining a colour-focused intervention block aligned with the scope of the review.

The supplementary validation search likewise used three concept blocks connected by the Boolean operators AND and OR, with database-specific syntax adaptations: (1) colour-related contextual terms; (2) major cognitive task-family terms, including visual search, attention, working memory, executive function, Stroop, n-back, cognitive load, and mental workload; and (3) outcome terms, including performance, accuracy, reaction time, response time, RT, and error-related terms. Unlike the primary search, the validation search did not require multimodal or sensor terms. Full database-specific search documentation for the supplementary validation search is likewise provided in the public repository as DataR6.

The same eligibility logic was applied in the supplementary validation search. Inclusion required: (a) explicit manipulation of colour as an independent variable; (b) the presence of a cognitive task or task-related performance context; and (c) at least one task-relevant outcome. Exclusion covered records without explicit in-scope colour manipulation, studies without a cognitive task or task-related outcome, records outside the predefined review scope, and non-empirical or otherwise ineligible designs. Reports already included in the original review set were not re-entered into the final synthesis.

The supplementary validation search identified 946 records, of which 265 were removed as duplicates, leaving 681 records for title and abstract screening. Sixty-four reports were sought for retrieval and underwent full-text assessment, with no reports unavailable for retrieval. Of these, 60 were excluded and 4 were newly included in the final synthesis. The main reasons for full-text exclusion in the validation pathway were outside the predefined review scope (*n* = 23), no cognitive task or outcome (*n* = 20), no explicit colour or lighting manipulation (*n* = 10), non-empirical or otherwise ineligible design (*n* = 4), and already included in the original review set (*n* = 3). The supplementary validation pathway identified only a limited number of newly eligible studies within the predefined review scope. This result improves transparency about the review’s retrieval boundaries and indicates that explicit task-family expansion could identify some additional relevant records, but it should not be interpreted as proof of exhaustive coverage under the original search structure. The study-selection pathway is summarised in Fig. 2.

### Data items and coding framework

Data extraction was pre-specified along the three CMC dimensions detailed in Sect. 3: Colour (manipulated parameter and locus, e.g. lightness, hue, chroma, colour difference, background, stimulus-background contrast, dynamic colour shift), Modality (behavioural, ocular, neurophysiological, autonomic, subjective), and Cognition (task family mapped to domain label, e.g. visual attention, working memory, executive function, logical reasoning, visuospatial construction).

Each study was additionally coded for participant characteristics, population type, rehab relevance tier, task type, colour parameterisation, device and ambient conditions where reported, modulation mode, and study-level direction of effect. For adaptive or closed-loop studies, engineering-related reporting items were also extracted, including signal source, update logic, latency or timescale information, stability safeguards, and comfort or accessibility constraints where reported.

Population type was classified as healthy-only, mixed, or clinical. Translational relevance was classified at three levels: Tier 1, direct clinical or rehabilitation-specific populations or settings; Tier 2, healthy-only or mixed populations performing rehab-proximal functional tasks; and Tier 3, general cognition, educational, or human-computer interaction contexts without direct rehabilitation targeting.

Direction-of-effect coding was based on the study authors’ reported results for the primary task and primary outcome. Positive indicated support for task performance or efficiency under the focal colour condition; negative indicated poorer performance or efficiency; null indicated no meaningful difference; and mixed or task-dependent indicated internally divergent findings across outcomes, conditions, or subgroups. Where multiple tasks or outcomes were reported, coding was anchored to the principal task family and the outcome most central to the study objective.

The core evidence matrix organised findings by cognitive domain, colour variable, locus of implementation, and measurement modality to support structured cross-study comparison. No pooled effect-size threshold was applied. Accordingly, the core evidence matrix was structured to support cross-study comparison of directional patterns rather than quantitative estimates of effect magnitude.

### Risk of bias assessment

The methodological quality and risk of bias for the 75 empirical studies were independently assessed by two reviewers. Given the heterogeneity of study designs, a suite of established critical appraisal tools was used: the Cochrane RoB 2 tool for randomised controlled trials (*n* = 14) [42], the ROBINS-I tool for non-randomised studies of interventions (*n* = 44) [43], the Newcastle-Ottawa Scale (NOS) for observational studies (*n* = 15) [44], and JBI Critical Appraisal Tools for other designs (*n* = 2) [45]. Any disagreements were resolved through discussion and consensus. The specific tool applied to each study and the detailed domain-by-domain results are reported in Supplementary Tables S2 and S3.

### Synthesis approach and qualitative certainty appraisal

Given the heterogeneity of tasks, colour parameters, populations, and outcomes, quantitative meta-analysis was not feasible. A structured narrative synthesis was therefore performed, combining (i) macro-level distributions, (ii) the core evidence matrix, and (iii) extraction of cross-cutting patterns. The primary extraction and coding dimensions are outlined in Table 1, and the complete coded dataset for all 75 studies is provided in the deposited extraction matrix and companion repository files (DataR1–DataR6).

This synthesis was informed by SWiM principles and used structured directional summaries rather than pooled effect-size estimates. Multimodal consistency was explicitly annotated to capture efficiency-related changes that may be masked by behavioural ceiling effects, while inconsistencies in colour parameter reporting were documented to characterise the field’s methodological limitations.

A formal outcome-level GRADE assessment was not feasible. Instead, a cluster-level qualitative evidence profile informed by the major GRADE domains was developed across risk of bias, inconsistency, indirectness, imprecision, and publication bias. This profile was used to guide interpretive confidence across outcome clusters and should not be interpreted as a formal GRADE rating of intervention effects. Cluster-specific certainty judgements are reported in DataR5. Translational interpretation was further constrained by population type and rehab relevance tier, such that implications were drawn within, rather than across, these strata. Formal quantitative assessment of publication bias was not appropriate because no pooled meta-analytic estimates were generated. Publication bias was therefore considered qualitatively through database coverage across the primary and supplementary searches, forward and backward citation tracking, the validation pathway specifically designed to relax modality-related retrieval constraints, and appraisal across GRADE-informed domains.

The review protocol was not prospectively registered. Eligibility criteria, extraction dimensions, and synthesis rules were specified before full-text coding, but no formal public registration record was created. The absence of prospective registration is therefore acknowledged as a review-level limitation.

**Table 1:** Data extraction and coding dimensions with representative examples

Extraction dimension	Definition	Representative examples
Bibliographic details	Core reference information	Author(s), year, country
Colour parameters	Parameter(s) defining the manipulation	Hue (h°), Lightness (L*), Chroma (C*), Colour difference (ΔE)
Modulation loci	Location and nature of colour manipulation	Background colour; Stimulus–background contrast; Dynamic colour shift
Cognitive domain	Primary cognitive function targeted in the task	Working memory (n-back), Visual attention (CPT/visual search), Executive function (Stroop), Logical reasoning (syllogistic reasoning), Visuospatial construction (mental rotation)
Measurement modality	Data type and measurement method	Behavioural (response time, accuracy); Neurophysiological (EEG, fNIRS); Ocular (e.g., fixation, pupil); Autonomic (HRV, EDA); Subjective (NASA-TLX)
Participant characteristics	Sample features relevant to interpretation and generalisability	Mean age, sex distribution, healthy adults, older adults, stroke, TBI, dyslexia, autism
Population type	Broad sample grouping used to stratify the evidence base	Healthy-only, mixed, clinical
Rehab relevance tier	Translational relevance of the study context	Tier 1: direct clinical or rehabilitation-specific population or setting; Tier 2: rehab-proximal functional task context; Tier 3: general cognition, educational, or human-computer interaction context
Effect direction	Study-level coded effect pattern	Positive (+), negative (-), mixed or task-dependent (±), null (0)
Modulation mode	Control regime for colour	Static (pre-set); reactive (rule-based or event-triggered); adaptive or closed-loop (signal-driven updating)
Device and ambient reporting	Hardware and environmental conditions reported for interpretation and reproducibility	Monitor, tablet, VR display, room lighting, ambient illumination, viewing distance, calibration information
Engineering reporting items	Additional implementation details extracted for adaptive or closed-loop studies	Signal source, update logic or decision rule, timing or latency, stability or tracking control, comfort or accessibility constraint

### 3. The Colour–Modality–Cognition (CMC) framework: a unified model for analysis and design

To address the challenges identified in the introduction, this section provides a detailed exposition of the unified analytical model specifically constructed for this research: the ‘Colour–Modality–Cognition’ (CMC) framework. The framework serves as the theoretical cornerstone for all subsequent evidence mapping and comprehensive discussion.

**Fig. 3:**
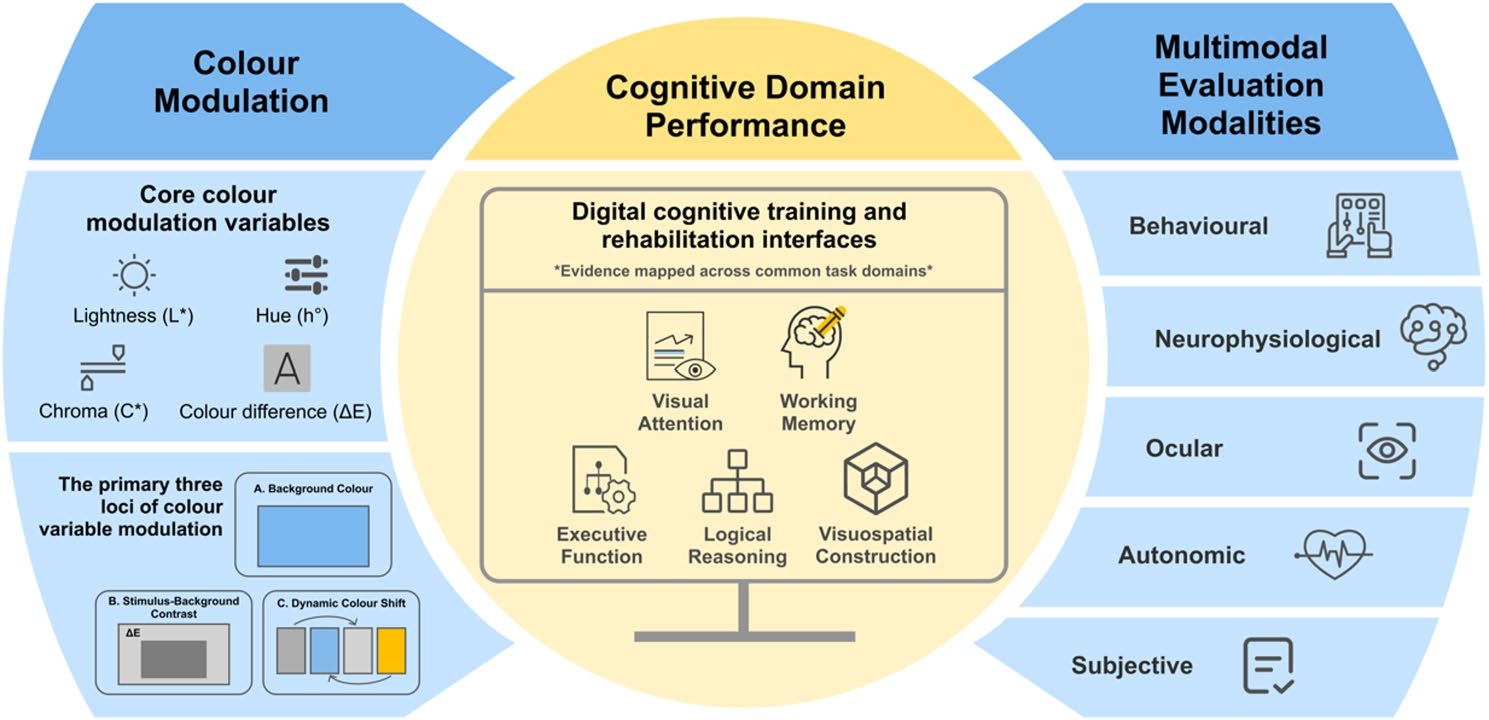
The Colour–Modality–Cognition (CMC) Framework

The fragmented nature of current research stems from the absence of a unifying, goal-oriented perspective to connect modulation tools, evaluation feedback, and final outcomes. The CMC framework is designed to establish this perspective, facilitating a strategic shift from the passive observation of colour effects to its active application as an intervention. As shown in Fig. 3, this framework treats the *Colour (the first C)* component as a parameterised intervention tool, *Modality (M)* as a state and outcome channel, and *Cognition Domain Performance (the second C)* as task-level performance to be supported.

The first component is *Colour (C)*, defined as a regulatory intervention tool that can be precisely controlled via standardised parameters. To ensure research reproducibility, the framework reconstructs colour variables within a perceptually uniform space (e.g., CIELAB) [46, 47]. The core variables for manipulation are Lightness (L*), Hue (h°), Chroma (C*), and Colour Difference (ΔE). As summarised in Table 2, each of these parameters corresponds to a distinct perceptual dimension that can be systematically adjusted to influence specific cognitive processes.

**Table 2:** Core colour modulation variables in a digital context

Variable	Definition in digital environment	Primary cognitive impact	Key application example	Unit
Lightness (L*)	Perceived screen brightness (0 = black, 100 = white) in CIELAB space.	Supports legibility and task reachability; is associated with alertness.	Optimising background L∗ to improve text readability [15, 19, 48–50].	CIELAB (0–100)
Hue (h°)	The pure colour attribute (e.g., red, blue) as an angle (0-360°) in CIE L*C*h° space.	Guides cognitive strategy and modulates affective state.	Using a blue background to support analytical tasks [9, 10, 18].	Degrees (°)
Chroma (C*)	Colour intensity/saturation relative to a neutral grey of the same lightness.	Regulates attentional capture and cognitive load.	Lowering interface C∗ to reduce distraction in memory tasks [19, 20, 29, 50]	CIELAB units
Colour Difference (ΔE)	Perceptual distance between two on-screen colours in CIELAB space.	Predicts stimulus discriminability, directly impacting accuracy and speed.	Maximising text-background ΔE to ensure legibility [15, 16, 51].	ΔE

**Fig. 4:**
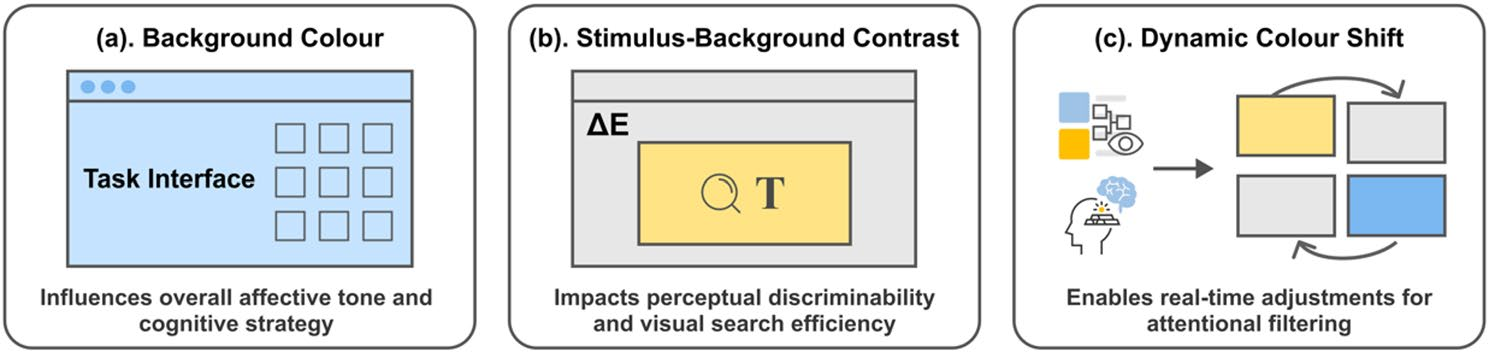
The primary three loci of colour variable modulation

These colour variables can be manipulated at three primary loci of modulation, as illustrated in Fig. 4. Each locus affects distinct cognitive processes: (a) Background Colour primarily influences overall affective tone and cognitive strategy [17–19]; (b) Stimulus-Background Contrast directly impacts perceptual discriminability and visual search efficiency [15, 16]; and (c) Dynamic Colour Shift enables real-time adjustments for attentional filtering based on user state [48–50].

The Modality (M) component aggregates how effects are detected and how future systems may sense user state. Five key pathways are categorised to distinguish overt task outcomes from latent internal states. They include: *behavioural (task performance)* measures (e.g., accuracy, response time) [25, 51]; *neurophysiological signals* (e.g., EEG, fNIRS) [52, 53]; *ocular signals* (e.g., fixation metrics and pupil diameter from eye-tracking) [31, 54]; *autonomic signals* (e.g., HRV, EDA) [19, 55]; and *subjective reports* (e.g., NASA-TLX scales) [17, 24]. This multimodal aggregation allows for a comprehensive assessment of both overt outcomes and latent cognitive load. Multimodal concordance is therefore central: when behaviour approaches a ceiling, ocular or neurophysiological markers often reveal efficiency-related changes that may inform colour adjustment and future adaptive control studies.

The *Cognition Domain Performance* (C) component represents the core target of the intervention: the cognitive performance that the adaptive rehabilitation system aims to optimise. To ensure that effects from heterogeneous studies can be evaluated consistently, the Cognition (C) component is defined as the task-level performance outcomes that the intervention aims to improve. These outcomes serve as the endpoint to judge whether colour modulation, sensed and guided via relevant modalities, improves the targeted function. For synthesis and reporting, tasks in the included studies are grouped into five functional domains: visual attention, working memory, executive function, logical reasoning, and visuospatial construction [32, 40, 56–59]. This grouping aligns evidence with the specific cognitive goals of rehabilitation tasks.

## Evidence synthesis and discussion

**Fig. 5:**
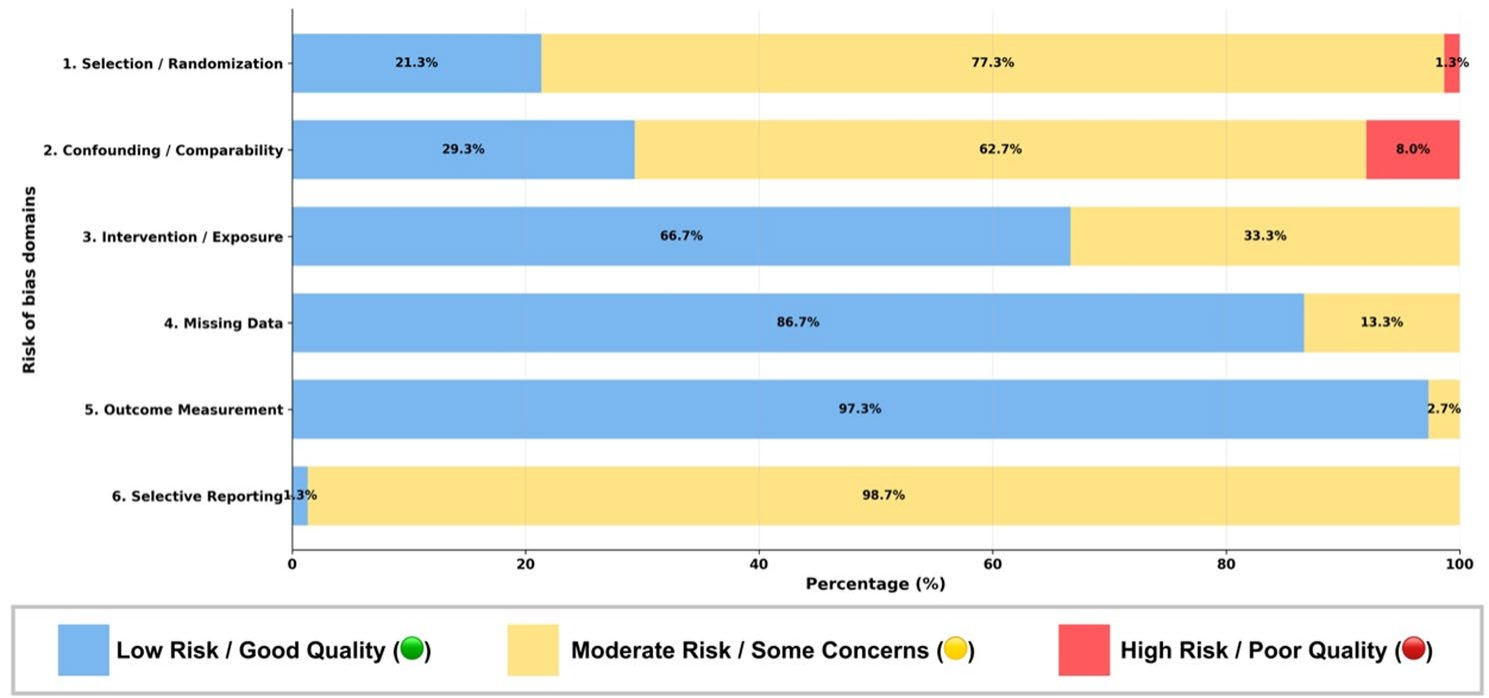
Summary of risk of bias assessment for included studies (*N* = 75). This summary is derived from the detailed study-by-study assessments provided in Supplementary Tables S2 and S3

Before synthesising the macro-level trends from the included literature, it is first necessary to establish the overall methodological quality of the evidence base. A methodological quality assessment of the 75 included studies indicated that risk was concentrated more in study selection, randomisation, and reporting-related domains than in outcome measurement itself (Fig. 5; detailed study-level judgements in Supplementary Tables S2 and S3). Low-risk judgements were more frequent for outcome measurement, intervention or exposure definition, and missing-data handling, whereas the most recurrent concerns related to limited preregistration, incomplete randomisation detail, and vulnerability to selective reporting. Because no pooled meta-analytic estimates were generated, formal quantitative assessment of publication bias was not appropriate. Publication bias was therefore considered qualitatively through database coverage across the primary and supplementary searches, forward and backward citation tracking, the validation pathway specifically designed to relax modality-related retrieval constraints, and its contribution to the cluster-level certainty appraisal. Residual bias favouring positive or more completely reported findings nevertheless cannot be excluded.

Applying the CMC lens to the 75 included studies reveals three measurable shifts that both validate the framework and highlight areas where the field remains underdeveloped. The ambiguous colour labels (e.g., “red/blue”) are increasingly replaced by parameterised reporting in a perceptually uniform space [15, 18, 60]. While Hue (h°) is the most frequently explored dimension across studies, the disclosure of standardised contrast parameters, namely Lightness (L*) and Colour Difference (ΔE), has become a central focus, reflecting a foundational emphasis on legibility [20]. By contrast, real-time, closed-loop dynamic colour control, where colour parameters update based on online state, remains rare and typically appears as a proof-of-concept rather than as a sustained training intervention [48–50, 61]. This asymmetry matters for translation: without closed-loop control, studies tend to capture immediate performance changes but under-sample post-training gains and retention.

**Fig. 6:**
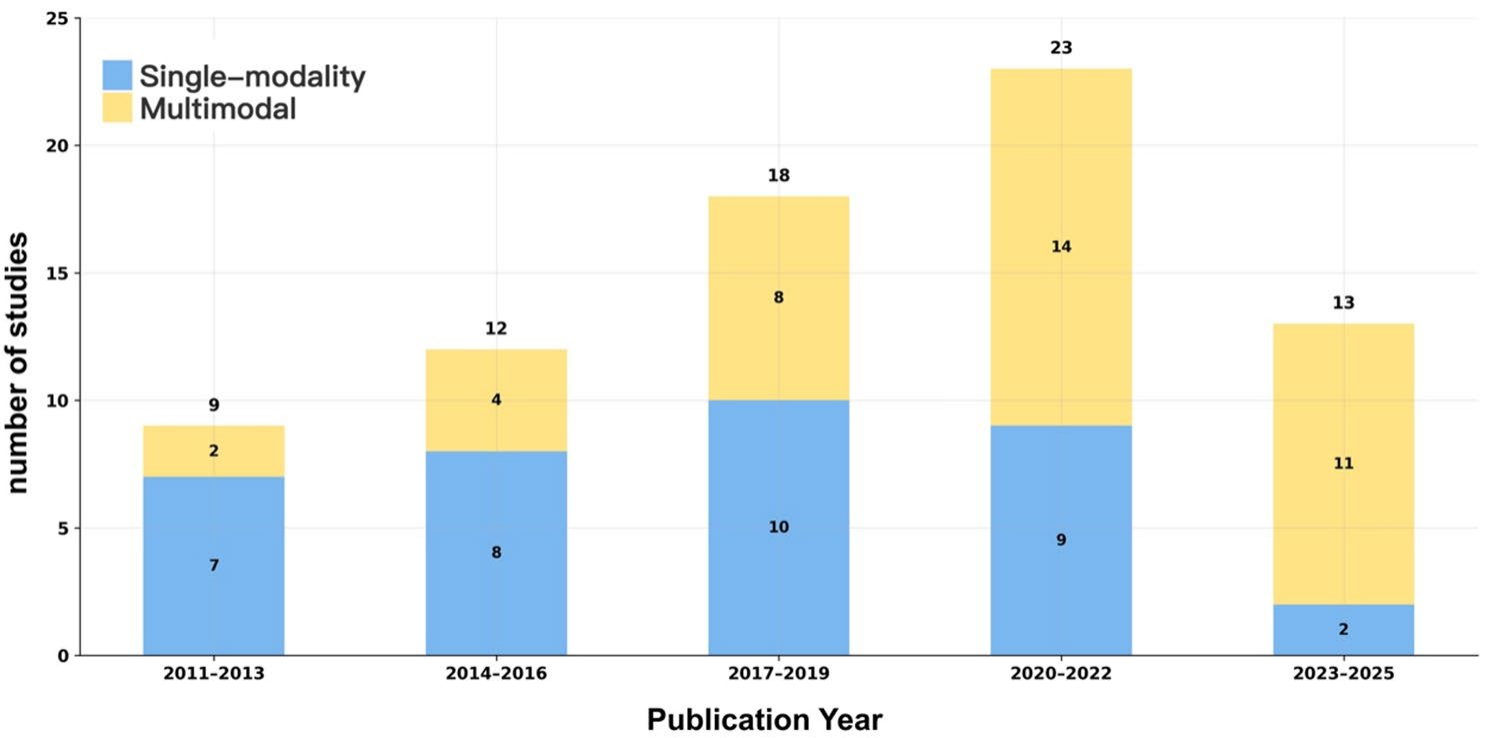
Distribution trend of single-modality versus multimodal studies by publication year. Single-modality studies are those employing a single measurement modality; multimodal studies employ two or more

The evaluation has shifted from single-modality endpoints to a convergent, multimodal assessment that pairs behavioural measures with ocular or neurophysiological markers. This shift, summarised in Fig. 6, is not cosmetic; it is necessary to detect efficiency-related changes that may be masked when behaviour saturates, for example, shorter first-fixation latencies, more economical scanpaths, or reduced prefrontal activation in the absence of further accuracy improvements. Multimodal readouts, therefore, act as early indicators that a configuration is helping users work more efficiently, even when classical task scores appear flat.

The evidence base remained unevenly distributed across cognitive domains, with studies focusing on visual attention (*n* = 21), working memory (*n* = 18), and executive function (*n* = 16), while logical reasoning and visuospatial construction remained under-sampled. This distribution motivates an evidence matrix (Table 3) that organises effects by domain, colour variable, and implementation context, enabling cross-domain distillation in the following subsection and anchoring the translational guidance thereafter. The certainty profile was likewise uneven. A qualitative certainty appraisal informed by GRADE domains indicated comparatively higher confidence for contrast-secured legibility and structured colour-cueing patterns, but low or very low confidence for hue- or chroma-based optimisation claims and closed-loop adaptive applications. This confidence gradient reflected recurrent indirectness relative to rehabilitation-specific populations and settings, inconsistency across task families and display conditions, imprecision arising from heterogeneous outcome structures, and residual concerns related to study-level bias and publication bias; cluster-level certainty judgements are reported in DataR5. The evidence base was dominated by predominantly healthy and non-clinical samples, with only a limited minority of studies providing direct clinical or rehabilitation-specific evidence. This composition supports domain-level synthesis but constrains rehabilitation-specific interpretation; detailed study-level stratification is provided in DataR2. Participant demographics were likewise heterogeneous across the evidence base, while age and sex reporting remained insufficiently consistent for a reliable pooled demographic summary. Any rehabilitation-oriented interpretation of the synthesis should therefore be read in light of this evidential composition, particularly the predominance of healthy-only samples and Tier 3 contexts, and not as equivalent to direct rehabilitation efficacy evidence. Against this background, the following sections distil the core cross-domain patterns emerging from the synthesis.

### Distillation of core patterns and interpretive implications

Within this evidence structure, Table 3 provides the domain-structured directional summary used for main-text interpretation, whereas DataR4 provides the complementary cross-cutting directional clusters that recur across domains.

**Table 3:** Structured directional summary of colour-modulation findings by cognitive domain, dominant sample composition, and translational context

Cognitive domain	Representative variables / loci	Structured directional summary	Sample and translational context	Interpretive note	Representative studies (R2 Ref #)
Visual attention	ΔE, L*, h°, C*; background colour; stimulus-background contrast; dynamic colour shift	More consistent support for contrast-secured legibility, local colour cueing, and adaptive highlighting in visual search. Background hue- and chroma-related effects were mixed or task-dependent.	Mostly healthy, with mixed and limited clinical representation; translational spread across Tiers 1 to 3, but predominantly Tier 3.	Evidence for rehabilitation remains indirect. Adjacent lighting-related evidence exists, but remains methodologically heterogeneous.	10, 23, 30, 35, 40, 43, 65, 68, 75
Working memory	L*, C*, h°; background colour; colour coding	Findings were mixed or task-dependent, varying with task design, baseline visual conditions, and whether manipulation was screen-based or ambient.	Healthy-only evidence in this synthesis subset; predominantly Tier 3 and indirect to rehabilitation-specific application.	Adjacent illuminance and CCT evidence suggests possible benefits under higher illuminance, whereas CCT effects remain inconsistent.	14, 32, 45, 47, 55, 58, 72, 73, 74
Executive function	ΔE, L*, h°; stimulus-background contrast; background colour; structured colour cues	More consistent support for structured colour coding and contrast-secured displays in applied contexts. Effects of background hue/chroma and lighting on inhibition remained mixed or task-dependent.	Mostly healthy, with sparse clinical representation; translational spread was predominantly Tier 3, with isolated Tier 1 and Tier 2 evidence.	Direct clinical evidence sparse. Interpretation should remain task-specific.	1, 11, 17, 21, 48, 50, 63, 73
Logical reasoning	h°, L*, C*; background colour	Evidence remains limited and task-dependent. Selected studies suggest possible benefits under controlled background settings, but findings are not yet consistent for stable inference.	Healthy-only and largely indirect; no direct rehabilitation-specific context in the present synthesis subset.	Evidence for rehabilitation remains indirect and is based on a small number of studies.	18, 19, 31
Visuospatial construction	ΔE, h°; stimulus-background contrast; colour coding	Preliminary support for contrast-secured displays and colour coding in spatial localisation, mental rotation, wayfinding, and navigation. Evidence remains context-dependent.	Healthy-only in the present subset; mainly applied non-clinical contexts, with limited Tier 2 functional-task relevance.	Benefits depend on task ecology and cue structure. Direct clinical translation remains underexplored.	16, 26, 34, 47, 51, 53

Table 3 presents a structured directional summary at the domain level rather than a pooled quantitative estimate. Effect-direction labels reflect study-level coding anchored to the principal task family and primary outcome of each study; mixed or task-dependent is used where findings diverged across outcomes, conditions, or subgroups. The Sample and translational context column summarises the dominant population composition and rehabilitation relevance represented within each domain cluster. Across the full dataset underpinning this synthesis, sample composition was 84.0% healthy-only, 9.3% mixed, and 6.7% clinical, while translational tiering comprised 10.7% Tier 1 direct clinical or rehabilitation studies, 14.7% Tier 2 rehabilitation-proximal studies, and 74.7% Tier 3 general cognition or HCI studies. R2 Ref # refers to the study identification number used in DataR2 (Population Stratification Matrix), not the manuscript reference list numbering. Accordingly, domain-level patterns in Table 3 should not be interpreted as carrying equivalent direct relevance to rehabilitation deployment across domains or study contexts

To support interpretation, certainty was appraised at the outcome-cluster level through a qualitative profile informed by risk of bias, inconsistency, indirectness, imprecision, and publication bias. A concise cluster-level certainty summary informed by these domains is incorporated into the interpretive framing of Table 3, while full cluster-level profiles are provided in DataR5. These domains were used to guide interpretive confidence rather than to generate formal GRADE ratings, given the heterogeneity of designs, outcomes, and synthesis structure. Study-level participant characteristics, clinical features, and translational tier assignments are reported in DataR2, engineering-reporting items in DataR3, and cross-cutting directional clusters in DataR4.

### Cross-domain graded and context-dependent patterns

Across the included clusters, a graded and context-dependent pattern was observed in the present directional synthesis. Within the present directional synthesis, lightness (L*) and colour difference (ΔE) were more consistently associated with perceptual discriminability and task reachability than hue (h°)- and chroma (C*)-based manipulations, particularly when contrast was the principal locus of implementation [15, 16, 34, 53]. Across several task families, contrast-secured displays were more often associated with improved legibility, faster responses, or reduced task difficulty, especially where baseline readability had not already reached saturation [15, 16, 51, 53].

Once baseline legibility was secured, hue- and chroma-related effects appeared more conditional on task family, implementation locus, and the contrast conditions already in place. Where baseline contrast was insufficient or where variable-locus combinations were poorly matched to task demands, additional hue- or chroma-based gains were not consistently observed. In some logical-reasoning contexts, cooler background hues were associated with more efficient performance, whereas in some working-memory contexts lower-chroma backgrounds were associated with reduced distraction or lower cognitive demand; however, these tendencies were observed only under restricted task and display conditions and should not be interpreted as domain-general optimisation rules [10, 17, 20, 62]. These patterns were not uniform across tasks, samples, or display settings, and high-chroma or poorly matched variable-locus combinations were more often associated with mixed findings [8, 9, 23]. Direct clinical representation nevertheless remained sparse across the evidence base.

Taken together, these findings support a cautious practical heuristic rather than a magnitude-based rule. A defensible design heuristic is to secure contrast and legibility first, and only then to consider domain-tuned h° and C* adjustments, with explicit attention to task family, implementation locus, baseline usability constraints, and the indirectness of much of the current evidence for rehabilitation-specific deployment.

### Hidden gains and the value of multimodal confirmation

A recurring implication of the present evidence base is that behavioural metrics alone may under-detect efficiency-related changes associated with colour modulation. When behavioural performance approaches ceiling levels, such as stable accuracy or response time, efficiency-related differences may become more apparent in ocular and neurophysiological measures, including shorter first-fixation latency, more economical scanpaths, and reduced prefrontal activation, which may indicate more efficient processing or lower cognitive load under some colour conditions [37, 39, 63–66]. Accordingly, behaviour-only endpoints may fail to capture potentially relevant changes in processing efficiency, whereas convergent multimodal readouts can provide complementary sensitivity for evaluation and adaptive control. Multimodal confirmation therefore provides an important complement for identifying these otherwise less visible efficiency-related changes.

### The critical role of modulation locus

The locus of modulation is an important design parameter. Three loci carry distinct mechanisms (Table 2), contrast chiefly targets discriminability (ΔE, L*) [11, 18, 53, 65], background tunes arousal/strategy (h°, C*) [17, 18, 67, 68], and dynamic shift enables state-contingent filtering [48–50, 69]. Reported gains were more often observed when the variable-locus pair matched the domain goal, for example, ΔE at contrast for attention and lower C* at background for working memory [64, 65]. This highlights a critical boundary condition: mismatched variable-locus pairs (e.g., high C* backgrounds in memory tasks) often yield mixed results [10, 11, 56].

### Translational guidance and design principles

The present synthesis supports a cautious translational framing organised around three methodological propositions: first, establish a contrast-secure baseline through lightness and colour difference conditions (L*, ΔE); second, consider conditional, domain-tuned hue and chroma adjustments (h°, C*); and third, evaluate potential efficiency-related changes alongside behavioural outcomes, particularly where behavioural performance approaches ceiling levels [21, 39, 64]. Given the current certainty profile, these principles should be interpreted as methodological and design-oriented recommendations rather than as outcome-grade clinical guidance.

The *Cognitive Performance Baseline* principle emphasises that the initial objective is to secure visual discernibility of informational elements. A consistent starting point is to prioritise lightness (L*) and contrast (ΔE), which showed more consistent support in the present synthesis for informational clarity and more efficient task processing [16, 51].

After securing the baseline, the *Gain Optimisation* principle involves domain-specific fine-tuning of hue (h°) and chroma (C*) to explore conditional additional gains. Unlike the more consistent support observed for L* and ΔE, this optimisation is not universal; additional gains should be expected only where baseline legibility has already been secured and where the selected h° or C* manipulation is plausibly aligned with the target task demands, implementation locus, and visual context [9, 11, 18]. This layered approach helps ensure that the pursuit of optimisation does not compromise fundamental usability.

Finally, the *Effect-Oriented* principle frames rehabilitation relevance as an outcome to be demonstrated rather than assumed. For rehabilitation-oriented applications, the value of colour modulation will depend not only on immediate in-task performance, but also on whether future studies demonstrate post-training retention, transfer, usability, adherence, and clinically meaningful benefit in appropriate populations and settings. This implies that a system’s closed-loop mechanism should leverage real-time multimodal data not just to maintain immediate in-task performance, but also to support learning conditions that may improve later outcomes, which remain insufficiently assessed in the current literature [48–50].

**Fig. 7:**
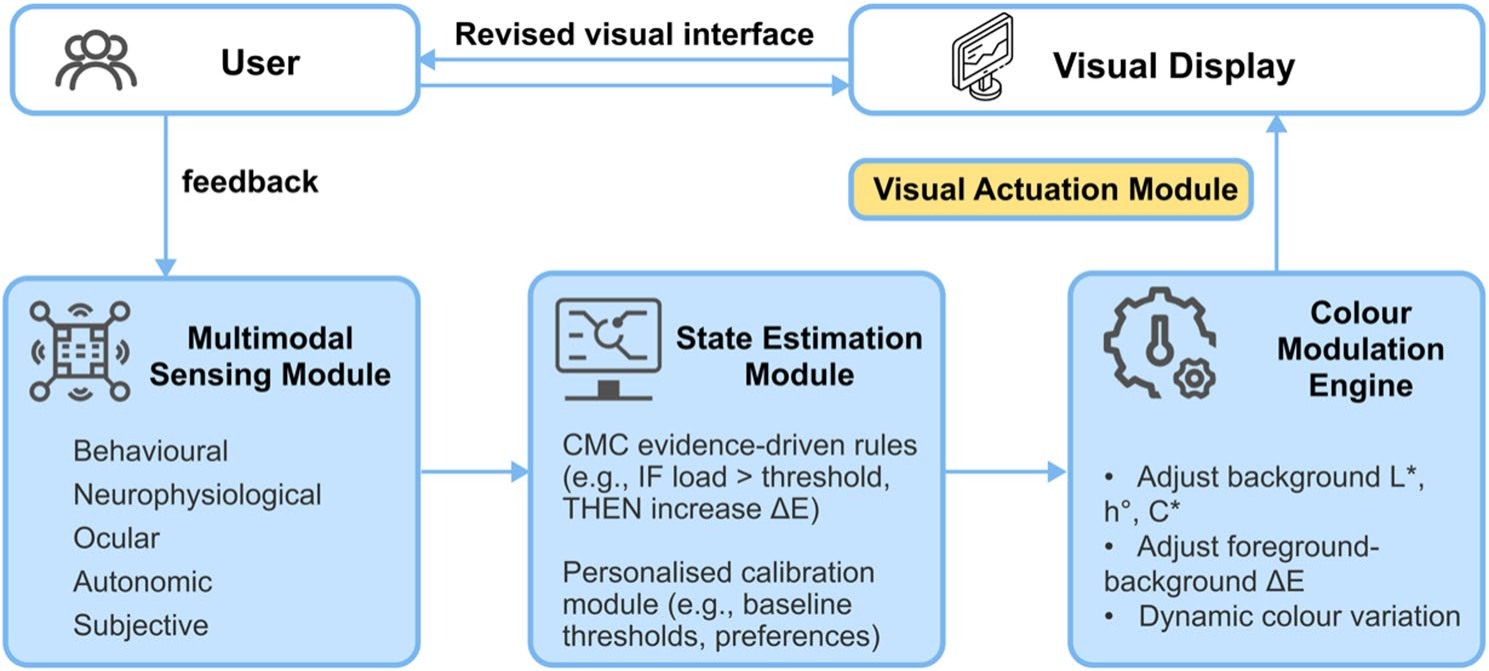
Core architecture of the closed-loop adaptive colour modulation system, showing auditable links between multimodal sensing, state estimation, colour-action logic, and visual actuation

Figure 7 operationalises these principles within a minimal, auditable architecture. This system comprises four core modules: *the Multimodal Sensing Module* (e.g., behavioural, ocular, neurophysiological data) to acquire raw state; *the State Estimation Module* to fuse indicators into a single efficiency-oriented score with explicit thresholds; *the Colour Modulation Engine* to map this state to colour actions under explicit safety constraints; and *the Visual Actuation Module* to apply these changes to the interface with complete metadata logging [70–73].

In practice, this architecture supports a simple, three-stage implementation pathway: (i) begin with a static, contrast-secure default condition; (ii) add reactive switching rules that move to a support palette when efficiency deteriorates; and (iii) progress to full closed-loop adaptation, where parameters are adjusted gradually based on the fused state estimate. This layered approach provides a structured pathway for rendering colour-modulation decisions more auditable and portable across studies and systems. Taken together, these items constitute a minimum reporting baseline for future colour-modulation studies, with additional control-method documentation required for any study claiming reactive, adaptive, or closed-loop updating. To support reproducibility and auditable implementation, future adaptive or gaze-contingent studies should report, at a minimum, the control regime, signal source, update logic, timing or latency characteristics, stability safeguards, and any comfort or accessibility constraints; these engineering-oriented reporting items are summarised in DataR3, alongside core display, calibration, and ambient-control descriptors.

### Key challenges and future research for implementing the CMC framework

Further development of the CMC framework for rehabilitation-oriented research requires that Colour, Modality, and Cognition be specified at the level of auditable procedures rather than high-level aspirations. To achieve this, the three challenges below are expressed as continuous prose so they can be implemented directly within the closed-loop pipeline shown in Fig. 7.

#### Challenge 1: Standardisation and personalisation in Colour (C)

Across the literature, reporting of colour parameters is inconsistent, and individuals often show different perceptual and affective responses to the same ΔE or h° [9–11, 30, 56]. The combination of heterogeneous reporting and genuine individual variability makes one-size-fits-all presets difficult to transfer across devices and contexts, weakening the accumulation of evidence across studies. A clear, auditable baseline in reporting is therefore important: at a minimum, disclose the foreground/background L* and ΔE, the h°/C* bands used, the device gamut and white point, and the calibration method, along with its update cadence [74]. On top of this baseline, a two-stage calibration provides a practical path: first, secure legibility and task reachability with mid-to-high L* and high ΔE; second, run a brief individual micro-tuning that adjusts h° and C* within predefined safety constraints, including avoidance of high-chroma backgrounds in memory-heavy tasks, avoidance of abrupt large-step colour transitions, and compatibility checks for common forms of colour-vision deficiency where colour discrimination is functionally relevant, using an efficiency score derived from ocular and neurophysiological markers as the optimisation criterion. This two-stage approach represents a practical solution to the standardisation-personalisation dilemma, creating a methodological balance: the standardised baseline ensures results are comparable across studies, while the personalised tuning may improve individual responsiveness [75]. The resulting personalised band should be written to metadata so that the policy logic within the State Estimation module in Fig. 7 can reuse it across sessions.

#### Challenge 2: Explainable fusion and latency-aware state estimation in Modality (M)

Closed-loop regulation requires state estimates that are sufficiently accurate, stable, and low-latency, derived from heterogeneous inputs such as behaviour, eye-tracking, and optional EEG/fNIRS [21, 48, 49, 64] In practice, fusion is often treated as a black box, which increases latency and reduces trust in clinical and engineering settings. A pragmatic default is to pair behaviour with at least one ocular or neurophysiological channel and to state explicitly the sampling rates, processing and decision latency, fusion rule (for example, a weighted score), thresholds, and any hysteresis or debounce logic [76, 77]. For practical implementation, a minimal sensor set such as behaviour and eye-tracking may provide a useful balance between cost, usability, and data quality [78, 79]. Methodologically, it is advisable to compare a lightweight, explainable scoring model (such as transparent rules or linear fusion) with a more complex learner under the same latency budget within the State Estimation module of Fig. 7. Co-primary endpoints can be decision latency and stability of the state estimate; secondary endpoints can include non-inferiority on the efficiency criterion and user or clinician interpretability ratings. If the explainable model meets non-inferiority under the latency constraint, it may be more feasible for rehabilitation-oriented closed-loop studies [80–83]. Furthermore, a critical challenge for future work is the development of intelligent fusion strategies to handle potential conflicts between modalities, for instance, when behavioural metrics suggest high performance while physiological signals indicate high cognitive load. Establishing clear conflict-resolution rules will be crucial for the robustness of the state estimation module [84, 85].

#### Challenge 3: Ecological validity and post-training gains in Cognition (C)

Most studies focus on immediate, in-task changes under controlled conditions, with limited observation of retention and transfer [10, 20, 25, 86]. Yet rehabilitation and education seek durable capability, not transient performance. Success may therefore be evaluated using a composite criterion: accuracy is non-inferior to baseline and at least one efficiency indicator (for example, first-fixation latency, scanpath economy, or reduced prefrontal load) improves. Protocols should pre-register a follow-up window and a set of near- and far-transfer tasks aligned with the target domain (see Table 3) [87, 88]. A practical design is a 12-week intervention with a 4 to 8 week follow-up, implemented through the Fig. 7 pipeline. The policy objective optimises the same composite criterion during training; primary endpoints at evaluation are the retention of efficiency gains and transfer to untrained tasks within the same domain family. In this way, ecological validity can be treated as a testable commitment along both temporal, retention, and task, transfer, dimensions [89, 90].

The three challenges above correspond to the critical interfaces of these modules, turning principles into testable components and reusable artefacts. The future agenda is threefold: standardise and personalise Colour in tandem, prefer explainable, latency-aware multimodal fusion, and redefine success around ecological validity and post-training gains. This positions the CMC framework as a more auditable methodological scaffold for future engineering and rehabilitation-oriented studies.

## Limitations

### Limitations of the present review

This review has several methodological limitations. First, the protocol was not prospectively registered, although eligibility criteria, extraction dimensions, and synthesis rules were specified before full-text coding. Second, substantial heterogeneity across tasks, colour manipulations, populations, and outcome measures precluded quantitative meta-analysis, and the synthesis therefore remained qualitative and directional rather than magnitude-based. Third, the review was restricted to peer-reviewed, English-language publications indexed in the selected databases and validation sources. Although forward and backward citation tracking and an additional validation search were used to reduce retrieval bias, grey literature was not formally incorporated into the synthesis. In addition, although the supplementary validation search broadened task-family terminology, the original primary search was not rerun under the expanded task-term structure, so some residual retrieval sensitivity cannot be excluded. Formal quantitative assessment of publication bias was therefore not appropriate, and residual risks of language bias, publication bias, and incomplete retrieval cannot be excluded. The resulting evidence base should therefore be interpreted as systematic within a defined review scope, but not as demonstrably exhaustive across the wider field.

### Limitations of the underlying evidence base

The underlying evidence base also has important limitations. Much of the included literature derives from general cognition, human-computer interaction, or experimentally controlled settings rather than from rehabilitation-specific populations or deployment contexts. Direct evidence remains limited for retention, transfer, adherence, and clinically meaningful benefit, which constrains the strength of rehabilitation-oriented inference. In addition, reporting of colour calibration, device characteristics, ambient conditions, and adaptive control logic was often incomplete, and many studies remained vulnerable to selective reporting, limited preregistration, or design-related confounding. These features limit comparability across studies and reduce certainty in stronger translational claims. Accordingly, the framework and guidance proposed here should be interpreted as a methodological research scaffold derived from the best available, but still incomplete and predominantly indirect, evidence.

## Conclusion

This review consolidates fragmented findings into a more coherent synthesis of how colour modulation has been studied across cognition, measurement, and adaptive control. Viewed through the Colour-Modality-Cognition framework, the evidence indicates a graded and context-dependent pattern: contrast-related variables, particularly L* and ΔE, showed more consistent support for baseline legibility and task reachability, whereas effects related to h° and C* were more conditional on task family, implementation locus, baseline contrast sufficiency, and the surrounding visual context, with less stable support where variable-locus combinations were poorly matched to task demands. Across studies, efficiency-related changes were often more readily detected in ocular and neurophysiological measures than in behavioural outcomes alone, particularly where performance approached ceiling levels.

On this basis, the review offers a cautious translational interpretation for rehabilitation-oriented research rather than a fixed rule for colour optimisation. Its principal contribution is a structured methodological scaffold linking colour variables, multimodal evaluation, and adaptive control logic in a form that is more auditable and comparable across studies. Current evidence therefore supports more explicit design and evaluation principles for rehabilitation-oriented colour research, particularly in relation to contrast-secured visual design and the use of multimodal assessment. However, translational implications should remain carefully bounded, as most available evidence derives from healthy-only or otherwise non-clinical task settings, with limited direct support for retention, transfer, adherence, or clinically meaningful benefit in rehabilitation-specific populations. The present framework should therefore be interpreted as a basis for more rigorous future rehabilitation research, not as evidence for routine implementation at the current stage.

## Supplementary Information

Below is the link to the electronic supplementary material.

Supplementary Material 1.

## Data Availability

The extracted study dataset, population stratification table, engineering-reporting matrix, structured directional synthesis summary, qualitative evidence profile, and full search documentation are available in Zenodo at [https://doi.org/10.5281/zenodo.18985161](https:/doi.org/10.5281/zenodo.18985161) as DataR1 to DataR6, respectively. The supplementary file provides the concise search overview, study-level risk-of-bias summary, detailed traffic-light plot, and bibliographic cross-reference list. No formal statistical analysis code was generated because the review did not involve quantitative meta-analysis or model-based computation.

## Abbreviations

C*: Chroma
CIELAB: A colour space specified by the International Commission on Illumination (representing Lightness, a*, and b* coordinates)
CMC: Colour–Modality–Cognition
CPT: Continuous performance test
ΔE: Colour difference
EDA: Electrodermal activity
EEG: Electroencephalography
fNIRS: Functional near-infrared spectroscopy
h°: Hue
HRV: Heart rate variability
JBI: Joanna Briggs institute
L*: Lightness
NASA-TLX: NASA task load index
NOS: Newcastle-Ottawa scale
PRISMA: Preferred reporting items for systematic reviews and meta-analyses
RoB 2: Cochrane risk of bias 2 tool
ROBINS-I: Risk of bias in non-randomised studies - of interventions

## References

[1] Frank Lopresti E, Mihailidis A, Kirsch N. Assistive technology for cognitive rehabilitation: State of the art. Neuropsychological rehabilitation. 2004;14(1–2):5–39.

[2] Irazoki E, Contreras-Somoza LM, Toribio-Guzmán JM, Jenaro-Río C, Van der Roest H, Franco-Martín MA. Technologies for cognitive training and cognitive rehabilitation for people with mild cognitive impairment and dementia. A systematic review. Front Psychol. 2020;11:648. 32373018 10.3389/fpsyg.2020.00648 PMC7179695

[3] Reinkensmeyer DJ. JNER at 15 years: analysis of the state of neuroengineering and rehabilitation. J Neuroeng Rehabil. 2019;16(1):144. 31744511 10.1186/s12984-019-0610-0 PMC6864952

[4] Cicerone KD, Langenbahn DM, Braden C, Malec JF, Kalmar K, Fraas M, et al. Evidence-based cognitive rehabilitation: updated review of the literature from 2003 through 2008. Arch Phys Med Rehabil. 2011;92(4):519–30. 21440699 10.1016/j.apmr.2010.11.015

[5] Ge S, Zhu Z, Wu B, McConnell ES. Technology-based cognitive training and rehabilitation interventions for individuals with mild cognitive impairment: a systematic review. BMC Geriatr. 2018;18(1):213. 30219036 10.1186/s12877-018-0893-1 PMC6139138

[6] Jovanov E, Milenkovic A, Otto C, De Groen PC. A wireless body area network of intelligent motion sensors for computer assisted physical rehabilitation. J Neuroeng Rehabil. 2005;2(1):6. 15740621 10.1186/1743-0003-2-6 PMC552302

[7] Elliot AJ, Maier MA. Color and psychological functioning. Curr Dir Psychol Sci. 2007;16(5):250–4.

[8] Makovski T, Swallow KM, Jiang YV. Attending to unrelated targets boosts short-term memory for color arrays. Neuropsychologia. 2011;49(6):1498–505. 21145331 10.1016/j.neuropsychologia.2010.11.029

[9] Xia G, Li M, Henry P, Westland S, Queiroz F, Peng Q, et al. Aroused and impulsive effects of colour stimuli on lateral and logical abilities. Behav Sci. 2021;11(2):24. 33562365 10.3390/bs11020024 PMC7916084

[10] Xia T, Song L, Wang TT, Tan L, Mo L. Exploring the effect of red and blue on cognitive task performances. Front Psychol. 2016;7:784. 27303343 10.3389/fpsyg.2016.00784 PMC4880552

[11] Wu J, Chen X, Zhao M, Xue C. Cognitive characteristics in wayfinding tasks in commercial and residential districts during daytime and nighttime: A comprehensive neuroergonomic study. Adv Eng Inform. 2024;61:102534.

[12] Lischinski D, Farbman Z, Uyttendaele M, Szeliski R. Interactive local adjustment of tonal values. ACM Trans Graphics (TOG). 2006;25(3):646–53.

[13] Dong M, Zhong L, editors. Chameleon: A color-adaptive web browser for mobile OLED displays. Proceedings of the 9th international conference on Mobile systems, applications, and services; 2011.

[14] Chou H-H, Nguyen A, Chortos A, To JW, Lu C, Mei J, et al. A chameleon-inspired stretchable electronic skin with interactive colour changing controlled by tactile sensing. Nat Commun. 2015;6(1):8011. 26300307 10.1038/ncomms9011 PMC4560774

[15] Wang X, Wang B, Xu L, Yu L. Tailored information display: Effects of background colour and line spacing on visual search across different character types–An eye-tracking study. Displays. 2025;88:103019.

[16] Lindquist LC, McIntire GR, Haigh SM. The effects of visual discomfort and chromaticity separation on neural processing during a visual task. Vision Res. 2021;182:27–35. 33588291 10.1016/j.visres.2021.01.007 PMC7987861

[17] Plass JL, Heidig S, Hayward EO, Homer BD, Um E. Emotional design in multimedia learning: Effects of shape and color on affect and learning. Learn Instruction. 2014;29:128–40.

[18] Jiang A, Gong Y, Yao X, Foing B, Allen R, Westland S, et al. Short-term virtual reality simulation of the effects of space station colour and microgravity and lunar gravity on cognitive task performance and emotion. Build Environ. 2023;227:109789.

[19] Wilms L, Oberfeld D. Color and emotion: effects of hue, saturation, and brightness. Psychol Res. 2018;82(5):896–914. 28612080 10.1007/s00426-017-0880-8

[20] Duan Y, Rhodes PA, Cheung V. T he influence of color on impulsiveness and arousal: P art 2–C hroma. Color Res Application. 2018;43(3):405–14.

[21] Bao J, Song X, Li Y, Bai Y, Zhou Q. Effect of lighting illuminance and colour temperature on mental workload in an office setting. Sci Rep. 2021;11(1):15284. 34315983 10.1038/s41598-021-94795-0 PMC8316362

[22] Yang C, Kong L, Zhang Z, Peng Y, Wang Q, Tao Y, et al. Research on cognition and inference model of interface color imagery based on EEG technology. Int J Human–Computer Interact. 2023;39(19):3774–85.

[23] Huettig F, Guerra E, Helo A. Towards understanding the task dependency of embodied language processing: The influence of colour during language-vision interactions. J Cognition. 2020;3(1):41. 10.5334/joc.135 PMC7583718 33134815

[24] Payedar-Ardakani P, Gorji-Mahlabani Y, Ghanbaran AH, Ebrahimpour R. Daylight illuminance levels, user preferences, and cognitive performance in office environments: Exploring an optimal illuminance range using virtual reality. Build Environ. 2024;258:111638.

[25] Hagmann CE, Wyble B, Shea N, LeBlanc M, Kates WR, Russo N. Children with autism detect targets at very rapid presentation rates with similar accuracy as adults. J Autism Dev Disord. 2016;46(5):1762–72. 26801777 10.1007/s10803-016-2705-9 PMC4826818

[26] Heuer A, Rolfs M. Predictable object motion is extrapolated to support visual working memory for surface features. Cognition. 2025;261:106150. 40306223 10.1016/j.cognition.2025.106150

[27] Musselman KE, Shah M, Zariffa J. Rehabilitation technologies and interventions for individuals with spinal cord injury: translational potential of current trends. J Neuroeng Rehabil. 2018;15(1):40. 29769082 10.1186/s12984-018-0386-7 PMC5956557

[28] Bonato P, Reinkensmeyer D, Manto M. Two decades of breakthroughs: charting the future of NeuroEngineering and Rehabilitation. Springer; 2025. pp. 1–2. 10.1186/s12984-025-01580-5 PMC11905428 40082907

[29] Kuzinas A, Noiret N, Bianchi R, Laurent É. The effects of image hue and semantic content on viewer’s emotional self-reports, pupil size, eye movements, and skin conductance response. Psychology of Aesthetics, Creativity, and the Arts. 2016;10(3):360.

[30] Vila-López N, Küster-Boluda I. Consumers’ physiological and verbal responses towards product packages: Could these responses anticipate product choices? Physiol Behav. 2019;200:166–73. 29510159 10.1016/j.physbeh.2018.03.003

[31] Wang Z-Y, Cho JY. Older adults’ response to color visibility in indoor residential environment using eye-tracking technology. Sensors. 2022;22(22):8766. 36433363 10.3390/s22228766 PMC9696812

[32] Roy S, Banerjee A, Roy C, Nag S, Sanyal S, Sengupta R, et al. Brain response to color stimuli: an EEG study with nonlinear approach. Cogn Neurodyn. 2021;15:1023–53. 34790269 10.1007/s11571-021-09692-z PMC8572309

[33] Zhang F, Wang X, Peng K, Xu L. Children’s physical fitness and cognitive control in China: the moderating role of family support for physical activity. BMC Public Health. 2025;25(1):1198. 40158184 10.1186/s12889-025-22397-w PMC11954196

[34] Mittelstädt S, Stoffel A, Keim DA, editors. Methods for compensating contrast effects in information visualization. Computer Graphics Forum. Wiley Online Library; 2014.

[35] Diachenko I, Kalishchuk S, Zhylin M, Kyyko A, Volkova Y. Color education: A study on methods of influence on memory. Heliyon. 2022;8(11). 10.1016/j.heliyon.2022.e11607 PMC9674548 36411932

[36] Stasenko A, Garcea FE, Dombovy M, Mahon BZ. When concepts lose their color: A case of object-color knowledge impairment. cortex. 2014;58:217–38. 25058612 10.1016/j.cortex.2014.05.013 PMC4135534

[37] Zhang M, Gong Y, Deng R, Zhang S. The effect of color coding and layout coding on users’ visual search on mobile map navigation icons. Front Psychol. 2022;13:1040533. 36582311 10.3389/fpsyg.2022.1040533 PMC9792669

[38] Liu K, Sun Z, Ren X, Tao D. Effects of information framing cues and age on the comprehension of personal health records for self-care behaviors: an eye-tracking study. J Am Med Inform Assoc. 2025;32(7):1174–85. 40460029 10.1093/jamia/ocaf085 PMC12203526

[39] Li Y, Fang W, Qiu H, Wang J. The non-visual effects of correlated color temperature on the alertness, cognition, and mood of fatigued individuals during the afternoon. Int J Ind Ergon. 2024;101:103589.

[40] Chen Y, Cheng W, Deng X, Yang Y, Li Z, Zhong J, et al. The influence of task-irrelevant color perception on flanker task performance: Insights from behavioral and ERP data. Physiol Behav. 2024;285:114654. 39111643 10.1016/j.physbeh.2024.114654

[41] Page MJ, McKenzie JE, Bossuyt PM, Boutron I, Hoffmann TC, Mulrow CD et al. The PRISMA 2020 statement: an updated guideline for reporting systematic reviews. BMJ. 2021;372. 10.1136/bmj.n71 PMC8005924 33782057

[42] Sterne JA, Savović J, Page MJ, Elbers RG, Blencowe NS, Boutron I et al. RoB 2: a revised tool for assessing risk of bias in randomised trials. BMJ. 2019;366. 10.1136/bmj.l4898 31462531

[43] Sterne JA, Hernán MA, Reeves BC, Savović J, Berkman ND, Viswanathan M et al. ROBINS-I: a tool for assessing risk of bias in non-randomised studies of interventions. BMJ. 2016;355. 10.1136/bmj.i4919 PMC5062054 27733354

[44] Wells GA, Shea B, O’Connell D, Peterson J, Welch V, Losos M et al. The Newcastle-Ottawa Scale (NOS) for assessing the quality of nonrandomised studies in meta-analyses. 2000.

[45] Munn Z, Barker TH, Moola S, Tufanaru C, Stern C, McArthur A, et al. Methodological quality of case series studies: an introduction to the JBI critical appraisal tool. JBI Evid synthesis. 2020;18(10):2127–33. 10.11124/JBISRIR-D-19-00099 33038125

[46] Safdar M, Cui G, Kim YJ, Luo MR. Perceptually uniform color space for image signals including high dynamic range and wide gamut. Opt Express. 2017;25(13):15131–51. 28788944 10.1364/OE.25.015131

[47] Mahy M, Van Eycken L, Oosterlinck A. Evaluation of uniform color spaces developed after the adoption of CIELAB and CIELUV. Color Res Application. 1994;19(2):105–21.

[48] Kunkel genannt Bode L, Schulte AS, Hauptmann B, Münte TF, Sprenger A, Machner B. Gaze-contingent display technology can help to reduce the ipsilesional attention bias in hemispatial neglect following stroke. J Neuroeng Rehabil. 2022;19(1):125. 36384816 10.1186/s12984-022-01104-5 PMC9670469

[49] Shen H, Asiry O, Babar MA, Bednarz T. Evaluating the efficacy of using a novel gaze-based attentive user interface to extend ADHD children’s attention span. Int J Hum Comput Stud. 2023;169:102927.

[50] Pan Y, Ge X, Ge L, Xu J. Using eye-controlled highlighting techniques to support both serial and parallel processing in visual search. Appl Ergon. 2021;97:103522. 34261002 10.1016/j.apergo.2021.103522

[51] Xia G, Henry P, Chen Y, Queiroz F, Westland S, Cheng Q. The effects of colour attributes on cognitive performance and intellectual abilities in immersive virtual environments. Comput Hum Behav. 2023;148:107853.

[52] Min BK, Kim HS, Ko W, Ahn MH, Suk HI, Pantazis D et al. Electrophysiological Decoding of Spatial and Color Processing in Human Prefrontal Cortex. NeuroImage. 2021;237. 10.1016/j.neuroimage.2021.118165 PMC8344402 34000400

[53] Takahashi N, Sawayama M, Chen X, Motomura Y, Takeichi H, Miyauchi S, et al. Temporal and spatial analysis of event-related potentials in response to color saliency differences among various color vision types. Front Hum Neurosci. 2024;18:1441380. 39416684 10.3389/fnhum.2024.1441380 PMC11479979

[54] Kress L, Bristle M, Aue T. Seeing through rose-colored glasses: How optimistic expectancies guide visual attention. PLoS ONE. 2018;13(2):e0193311. 29466420 10.1371/journal.pone.0193311 PMC5821386

[55] Lasauskaite R, Cajochen C. Influence of lighting color temperature on effort-related cardiac response. Biol Psychol. 2018;132:64–70. 29133144 10.1016/j.biopsycho.2017.11.005

[56] Nissen A, editor. editor Psychological and Physiological Effects of Color Use on eCommerce Websites: a Neural Study Using fNIRS. ICIS; 2020.

[57] Kooiker MJ, Pel JJ, van der Steen-Kant SP, van der Steen J. A method to quantify visual information processing in children using eye tracking. J visualized experiments: JoVE. 2016;113:54031. 10.3791/54031 PMC4993407 27500922

[58] Lian X, Hong WCH, Xu X, Kimberly K-Z, Wang Z. The influence of picture book design on visual attention of children with autism: a pilot study. Int J Dev Disabil. 2023;69(6):946–56. 37885844 10.1080/20473869.2022.2033590 PMC10599195

[59] Ooms K, De Maeyer P, Fack V. Study of the attentive behavior of novice and expert map users using eye tracking. Cartography Geographic Inform Sci. 2014;41(1):37–54.

[60] Xu L, Jia D, Zhang Z, Huang L, Xia G, Yu L, editors. The effect of reading background colour on human cognitive performance based on multi-modal data analysis-A study of gender differences. Proceedings of the Midterm Meeting of the International Colour Association; 2024.

[61] Mauderer M, Flatla DR, Nacenta MA, editors. Gaze-contingent manipulation of color perception. Proceedings of the 2016 CHI Conference on Human Factors in Computing Systems; 2016.

[62] Jue J, Kwon S-M. Does colour say something about emotions? Laypersons’ assessments of colour drawings. Arts Psychother. 2013;40(1):115–9.

[63] Zhang L, Li X, Li C, Zhang T. Research on visual comfort of color environment based on the eye-tracking method in subway space. J Building Eng. 2022;59:105138.

[64] Wang X, Xu L, Li J, Cheng C, Yu L. How Background Colour Shapes Digital Text-Information Processing: A fNIRS-Eye Tracking Study. Int J Human–Computer Interact. 2025:1–15.

[65] Xu L, Hou B, Wang X, Zhang Z, Yu L. Exploring the effects of background colour and gender on cognitive performance of visual attention: a multimodal approach using fNIRS and eye-tracking. J Int Colour Association. 2025;38:30–40.

[66] Laxton V, Maratos FA, Hewson DW, Baird A, Stupple EJ. Standardised colour-coded compartmentalised syringe trays improve anaesthetic medication visual search and mitigate cognitive load. Br J Anaesth. 2023;130(3):343–50. 36801016 10.1016/j.bja.2022.11.012 PMC10061295

[67] Dianat I, Sedghi A, Bagherzade J, Jafarabadi MA, Stedmon AW. Objective and subjective assessments of lighting in a hospital setting: implications for health, safety and performance. Ergonomics. 2013;56(10):1535–45. 23879884 10.1080/00140139.2013.820845

[68] Ho M-C, Chen J-M, Huang R-Y, Shen M-H, Lu M-C, Liu C-J. Numerical analysis on color preference and visual comfort from eye tracking technique. Math Probl Eng. 2015;2015(1):861610.

[69] Wang Q, Sun M, Liu H, Pan Y, Wang L, Ge L. The applicability of eye-controlled highlighting to the field of visual searching. Australian J Psychol. 2018;70(3):294–301. 30197433 10.1111/ajpy.12200 PMC6120491

[70] Chen M, Herrera F, Hwang K. Cognitive computing: architecture, technologies and intelligent applications. Ieee Access. 2018;6:19774–83.

[71] Ramsey NF, Van De Heuvel MP, Kho KH, Leijten FS. Towards human BCI applications based on cognitive brain systems: an investigation of neural signals recorded from the dorsolateral prefrontal cortex. IEEE Trans Neural Syst Rehabil Eng. 2006;14(2):214–7. 16792297 10.1109/TNSRE.2006.875582

[72] Lahat D, Adali T, Jutten C. Multimodal data fusion: an overview of methods, challenges, and prospects. Proc IEEE. 2015;103(9):1449–77.

[73] Gao J, Li P, Chen Z, Zhang J. A survey on deep learning for multimodal data fusion. Neural Comput. 2020;32(5):829–64. 32186998 10.1162/neco_a_01273

[74] Akkaynak D, Treibitz T, Xiao B, Gürkan UA, Allen JJ, Demirci U, et al. Use of commercial off-the-shelf digital cameras for scientific data acquisition and scene-specific color calibration. J Opt Soc Am A. 2014;31(2):312–21. 10.1364/JOSAA.31.000312 PMC4028365 24562030

[75] Reinecke K, Bernstein A. Improving performance, perceived usability, and aesthetics with culturally adaptive user interfaces. ACM Trans Computer-Human Interact (TOCHI). 2011;18(2):1–29.

[76] Xu J, Bao T, Lee UH, Kinnaird C, Carender W, Huang Y, et al. Configurable, wearable sensing and vibrotactile feedback system for real-time postural balance and gait training: proof-of-concept. J Neuroeng Rehabil. 2017;14(1):102. 29020959 10.1186/s12984-017-0313-3 PMC5637356

[77] Wang Q, Markopoulos P, Yu B, Chen W, Timmermans A. Interactive wearable systems for upper body rehabilitation: a systematic review. J Neuroeng Rehabil. 2017;14(1):20. 28284228 10.1186/s12984-017-0229-y PMC5346195

[78] Corcoran PM, Nanu F, Petrescu S, Bigioi P. Real-time eye gaze tracking for gaming design and consumer electronics systems. IEEE Trans Consum Electron. 2012;58(2):347–55.

[79] Novák JŠ, Masner J, Benda P, Šimek P, Merunka V. Eye tracking, usability, and user experience: A systematic review. Int J Human–Computer Interact. 2024;40(17):4484–500.

[80] Molnar C. Interpretable machine learning: Lulu. com; 2020.

[81] Murdoch WJ, Singh C, Kumbier K, Abbasi-Asl R, Yu B. Definitions, methods, and applications in interpretable machine learning. Proc Natl Acad Sci. 2019;116(44):22071–80. 31619572 10.1073/pnas.1900654116 PMC6825274

[82] Saraswat D, Bhattacharya P, Verma A, Prasad VK, Tanwar S, Sharma G, et al. Explainable AI for healthcare 5.0: opportunities and challenges. IEEe Access. 2022;10:84486–517.

[83] Chaddad A, Peng J, Xu J, Bouridane A. Survey of explainable AI techniques in healthcare. Sensors. 2023;23(2):634. 36679430 10.3390/s23020634 PMC9862413

[84] Hu Z, Lou S, Xing Y, Wang X, Cao D, Lv C. Review and perspectives on driver digital twin and its enabling technologies for intelligent vehicles. IEEE Trans Intell Veh. 2022;7(3):417–40.

[85] Zhang Y, Tu C, Gao K, Wang L. Multisensor information fusion: Future of environmental perception in intelligent vehicles. J Intell Connected Veh. 2024.

[86] Daniels EC, Rodriguez A, Zabelina DL. Severity of misophonia symptoms is associated with worse cognitive control when exposed to misophonia trigger sounds. PLoS ONE. 2020;15(1):e0227118. 31945068 10.1371/journal.pone.0227118 PMC6964854

[87] Perkins DN, Salomon G. Transfer of learning. Int encyclopedia Educ. 1992;2(2):6452–7.

[88] Ellis HC. The transfer of learning. 1965.

[89] Chaytor N, Schmitter-Edgecombe M. The ecological validity of neuropsychological tests: A review of the literature on everyday cognitive skills. Neuropsychol Rev. 2003;13(4):181–97. 15000225 10.1023/b:nerv.0000009483.91468.fb

[90] Spooner DM, Pachana NA. Ecological validity in neuropsychological assessment: A case for greater consideration in research with neurologically intact populations. Arch Clin Neuropsychol. 2006;21(4):327–37. 16769198 10.1016/j.acn.2006.04.004

