# Supplementary material for: Multimodal colour modulation for cognitive enhancement in intelligent rehabilitation: a systematic review and translational guidance
Source: J Neuroeng Rehabil. 2026 May 11;23:208. doi: 10.1186/s12984-026-01991-y (PMC13335281; doi:10.1186/s12984-026-01991-y)
Supplement: Supplementary file 1 — Supplementary Material 1. [file 12984_2026_1991_MOESM1_ESM.docx]

**Supplementary Materials**

This document provides supplementary materials for the manuscript titled: ***"Multimodal Colour Modulation for Cognitive Enhancement in Intelligent Rehabilitation: A Systematic Review and Translational Guidance".***

This supplementary document provides the search overview, risk-of-bias materials, and bibliographic cross-reference information that directly support the main text. Extended extraction, population stratification, engineering-reporting, structured directional synthesis, qualitative evidence profile, and full search-documentation files are available in Zenodo at <https://doi.org/10.5281/zenodo.18985161> as DataR1 to DataR6, respectively.

**1. Primary and supplementary validation search strategies**

The primary search was concept-driven and focused on colour-cognition studies indexed in Web of Science, Scopus, and PubMed, with Google Scholar citation tracking used to extend retrieval around key records. A supplementary validation search was subsequently conducted to test the coverage of the primary search and reduce potential retrieval bias arising from modality-specific terms. Database-specific syntax was adapted as required. Unlike the primary search, the supplementary validation search did not require multimodal or sensor terms. A concise overview of both search layers is provided in Supplementary Table S1, and the full database-specific search documentation is deposited in the public repository as DataR6.

**Supplementary Table S1.** Search overview and concise database search summary

| **Search layer** | **Databases** | **Concept blocks** | **Restrictions / notes** | **Full documentation location** |
| --- | --- | --- | --- | --- |
| Primary concept-driven search | Web of Science, Scopus, PubMed, plus Google Scholar citation tracking | Colour manipulation terms; cognition- or task-related terms; translational and modality-related terms aligned with the original concept-driven review scope | Peer-reviewed empirical studies; database-specific syntax adapted as required; multimodal or sensor-related framing retained in accordance with the original review design | Public repository, DataR6 |
| Supplementary validation search | Web of Science, Scopus, PubMed, APA PsycINFO, IEEE Xplore | colour-related contextual terms; major cognitive task-family terms; performance or outcome terms | Designed as a coverage check on the primary search; expanded task-family retrieval; multimodal or sensor terms were not required; grey literature, trial registries, and secondary syntheses were not incorporated into the formal search framework | Public repository, DataR6 |

**Note.** This table provides a concise overview of the two search layers used in the review. Full database-specific search strings, syntax adaptations, and search documentation are deposited in the public repository as DataR6.

**2. Risk of Bias Assessment**

A formal risk-of-bias assessment was conducted for the 75 empirical studies included in the synthesis. Owing to study-design heterogeneity, different appraisal tools were applied as appropriate: RoB 2 for randomised controlled trials (n = 14), ROBINS-I for non-randomised intervention-type studies, including within-subject and quasi-experimental designs involving active colour-modulation conditions without randomised parallel allocation (n = 44), the Newcastle-Ottawa Scale for observational studies (n = 15), and JBI Critical Appraisal Tools for other designs (n = 2).

**Supplementary Table S2.** Summary of Risk of Bias Assessment for Each Included Study

| **Ref#** | **Author(s), year** | **Study Design** | **Tool** | **Assessment Summary / Justification** | **Overall Judgment** |
| --- | --- | --- | --- | --- | --- |
| 1 | Payedar-Ardakani et al. (2024) | Within-subject Exp. | ROBINS-I | Moderate risk: Order randomized, but potential for time-varying confounders (fatigue/learning). | Moderate Risk |
| 2 | Jue & Kwon (2013) | RCT | RoB 2 | Some concerns: Randomization method not detailed; blinding impossible. | Some concerns |
| 3 | Dianat et al. (2013) | Observational | NOS | Fair sample selection but no control for key confounders. (6/9 stars) | Fair Quality |
| 4 | Plass et al. (2014) | RCT | RoB 2 | Some concerns: Randomization details lacking; blinding impossible; post-hoc data exclusion. | Some concerns |
| 5 | Rouw & Scholte (2016) | Observational | NOS | Groups are naturally occurring; comparability is not controlled for. (6/9 stars) | Fair Quality |
| 6 | Daniels et al. (2020) | Within-subject Exp. | ROBINS-I | Moderate risk: Carryover effects between sound conditions are possible; no pre-registration. | Moderate Risk |
| 7 | Stasenko et al. (2014) | Case Study | JBI | High-quality, detailed report meeting all criteria for a case study. (8/8 items) | Good Quality |
| 8 | Vila-López & Küster-Boluda (2019) | RCT (Crossover) | RoB 2 | Some concerns: Blinding impossible; no pre-registration mentioned. | Some concerns |
| 9 | Martin et al. (2015) | Qualitative Study | JBI | Strong methodological congruence and clear analytical process. (10/10 items) | Good Quality |
| 10 | Hagmann et al. (2016) | Observational | NOS | Good case-control matching on key variables (age, IQ). (8/9 stars) | Good Quality |
| 11 | Hartstein et al. (2018) | RCT | RoB 2 | Some concerns: Post-hoc exclusion of participants; randomization details lacking. | Some concerns |
| 12 | Zhang et al. (2025) | Observational | NOS | Good sample selection but limited control over confounding variables. (6/9 stars) | Fair Quality |
| 13 | Hu et al. (2025) | Observational | NOS | Clear case/control definitions and good comparability on age. (7/9 stars) | Good Quality |
| 14 | Heuer & Rolfs (2025) | Within-subject Exp. | ROBINS-I | Moderate risk: Order effects are a potential confounder, though trial order was randomized. | Moderate Risk |
| 15 | Xia et al. (2016) | RCT | RoB 2 | Some concerns: Blinding impossible; post-hoc data exclusion based on performance. | Some concerns |
| 16 | Mittelstädt et al. (2014) | RCT (Crossover) | RoB 2 | Some concerns: Blinding of perceptual task is impossible; no pre-registration. | Some concerns |
| 17 | Duan et al. (2018) | Within-subject Exp. | ROBINS-I | Moderate risk: Potential for learning/fatigue effects; no pre-registration. | Moderate Risk |
| 18 | Xia et al. (2021) | Within-subject Exp. | ROBINS-I | Moderate risk: Potential for learning/fatigue effects; no pre-registration. | Moderate Risk |
| 19 | Xia et al. (2023) | Within-subject Exp. | ROBINS-I | Moderate risk: Potential for learning/fatigue effects; no pre-registration. | Moderate Risk |
| 20 | Makovski et al. (2011) | RCT | RoB 2 | Some concerns: Post-hoc data exclusion based on accuracy; randomization details lacking. | Some concerns |
| 21 | Årdal & Hammar (2011) | Observational | NOS | Prospective cohort design with clear follow-up, but some loss to follow-up. (7/9 stars) | Good Quality |
| 22 | Diachenko et al. (2022) | Non-randomized Exp. | ROBINS-I | High risk: Non-random assignment of pre-existing groups (classes) introduces high risk of confounding. | High Risk |
| 23 | Wang et al. (2025) | Within-subject Exp. | ROBINS-I | Moderate risk due to potential time-varying confounders and lack of pre-registration. | Moderate Risk |
| 24 | Zhang et al. (2022) | Within-subject Exp. | ROBINS-I | Moderate risk due to potential time-varying confounders and lack of pre-registration. | Moderate Risk |
| 25 | Ho et al. (2015) | Within-subject Exp. | ROBINS-I | Moderate risk due to potential time-varying confounders and lack of pre-registration. | Moderate Risk |
| 26 | Li et al. (2025) | Within-subject Exp. | ROBINS-I | Moderate risk due to potential time-varying confounders and lack of pre-registration. | Moderate Risk |
| 27 | Kress et al. (2018) | RCT | RoB 2 | Some concerns: Blinding impossible; data exclusion based on behavior. | Some concerns |
| 28 | Lee & Chen (2025) | Within-subject Exp. | ROBINS-I | Moderate risk due to potential time-varying confounders and lack of pre-registration. | Moderate Risk |
| 29 | Lian et al. (2023) | Observational | NOS | Good case-control matching; objective outcome measure (eye-tracking). (8/9 stars) | Good Quality |
| 30 | Wang & Cho (2022) | Within-subject Exp. | ROBINS-I | Moderate risk: Order randomized, but potential for time-varying confounders. | Moderate Risk |
| 31 | Huettig et al. (2020) | Within-subject Exp. | ROBINS-I | Moderate risk: Potential for learning/fatigue effects across task domains. | Moderate Risk |
| 32 | Ooms et al. (2014) | Observational | NOS | Compares pre-existing groups (novice/expert) without controlling for confounders. (5/9 stars) | Fair Quality |
| 33 | Lindquist et al. (2021) | Within-subject Exp. | ROBINS-I | Moderate risk due to potential time-varying confounders and lack of pre-registration. | Moderate Risk |
| 34 | Min et al. (2021) | Within-subject Exp. | ROBINS-I | Moderate risk due to potential time-varying confounders and lack of pre-registration. | Moderate Risk |
| 35 | Takahashi et al. (2024) | Within-subject Exp. | ROBINS-I | Moderate risk due to potential time-varying confounders and lack of pre-registration. | Moderate Risk |
| 36 | Chai & Tang (2023) | Within-subject Exp. | ROBINS-I | Moderate risk due to potential time-varying confounders and lack of pre-registration. | Moderate Risk |
| 37 | Sarangi et al. (2021) | Within-subject Exp. | ROBINS-I | Moderate risk due to potential time-varying confounders and lack of pre-registration. | Moderate Risk |
| 38 | Chai et al. (2019) | Within-subject Exp. | ROBINS-I | Moderate risk due to potential time-varying confounders and lack of pre-registration. | Moderate Risk |
| 39 | Hasegawa et al. (2025) | Within-subject Exp. | ROBINS-I | Moderate risk due to potential time-varying confounders and lack of pre-registration. | Moderate Risk |
| 40 | M. Zhang et al. (2022) | Within-subject Exp. | ROBINS-I | Moderate risk due to potential time-varying confounders and lack of pre-registration. | Moderate Risk |
| 41 | Jin et al. (2022) | Within-subject Exp. | ROBINS-I | Moderate risk due to potential time-varying confounders and lack of pre-registration. | Moderate Risk |
| 42 | Liu et al. (2025) | RCT | RoB 2 | Some concerns: Randomization details lacking; age is also an observational factor. | Some concerns |
| 43 | Fang et al. (2025) | Within-subject Exp. | ROBINS-I | Moderate risk due to potential time-varying confounders and lack of pre-registration. | Moderate Risk |
| 44 | Lewandowska & Olejnik-Krugly (2022) | RCT | RoB 2 | Some concerns: Randomization details lacking; blinding impossible. | Some concerns |
| 45 | Wang et al. (2025) | Within-subject Exp. | ROBINS-I | Moderate risk due to potential time-varying confounders and lack of pre-registration. | Moderate Risk |
| 46 | Xu et al. (2025) | Non-randomized Exp. | ROBINS-I | Moderate risk: Gender is an observational factor; color is within-subject. Potential confounding. | Moderate Risk |
| 47 | Xu et al. (2024) | Observational | NOS | Compares gender groups; lacks control for potential confounders. (5/9 stars) | Fair Quality |
| 48 | Semin et al. (2018) | Observational | NOS | Compares gender groups; lacks control for potential confounders. (5/9 stars) | Fair Quality |
| 49 | Matzen et al. (2021) | Within-subject Exp. | ROBINS-I | Moderate risk due to potential time-varying confounders and lack of pre-registration. | Moderate Risk |
| 50 | Li et al. (2024) | RCT (Crossover) | RoB 2 | Some concerns: Blinding difficult; randomization details lacking. | Some concerns |
| 51 | Wu et al. (2024) | Within-subject Exp. | ROBINS-I | Moderate risk due to potential time-varying confounders and lack of pre-registration. | Moderate Risk |
| 52 | Baudry & Gaillard (2014) | Observational | NOS | Compares age groups and tasks; limited control for confounders. (6/9 stars) | Fair Quality |
| 53 | Kalantari et al. (2021) | Within-subject Exp. | ROBINS-I | Moderate risk due to potential time-varying confounders and lack of pre-registration. | Moderate Risk |
| 54 | Lim et al. (2025) | Observational | NOS | Case-control design (Bipolar vs HC) with good group comparability. (7/9 stars) | Good Quality |
| 55 | Bao et al. (2021) | Within-subject Exp. | ROBINS-I | Moderate risk due to potential time-varying confounders and lack of pre-registration. | Moderate Risk |
| 56 | Nissen et al. (2020) | Non-randomized Exp. | ROBINS-I | Moderate risk: Gender is an observational factor; color is within-subject. Potential confounding. | Moderate Risk |
| 57 | Folgieri et al. (2013) | Within-subject Exp. | ROBINS-I | Moderate risk due to potential time-varying confounders and lack of pre-registration. | Moderate Risk |
| 58 | Jiang et al. (2023) | RCT | RoB 2 | Some concerns: Small sample size per group; randomization details lacking. | Some concerns |
| 59 | Hawes et al. (2012) | RCT | RoB 2 | Some concerns: Blinding impossible; randomization details lacking. | Some concerns |
| 60 | Kuzinas et al. (2016) | Within-subject Exp. | ROBINS-I | Moderate risk due to potential time-varying confounders and lack of pre-registration. | Moderate Risk |
| 61 | Wilms & Oberfeld (2018) | Within-subject Exp. | ROBINS-I | Moderate risk due to potential time-varying confounders and lack of pre-registration. | Moderate Risk |
| 62 | Jakovljević et al. (2021) | Observational | NOS | Case-control design (Dyslexia vs HC) with good group matching. (8/9 stars) | Good Quality |
| 63 | Laxton et al. (2023) | Within-subject Exp. | ROBINS-I | Moderate risk due to potential time-varying confounders and lack of pre-registration. | Moderate Risk |
| 64 | Lasauskaite & Cajochen (2017) | RCT | RoB 2 | Some concerns: Randomization details lacking; blinding difficult. | Some concerns |
| 65 | Mauderer et al. (2016) | Within-subject Exp. | ROBINS-I | Moderate risk due to potential time-varying confounders and lack of pre-registration. | Moderate Risk |
| 66 | Machner et al. (2020) | Within-subject Exp. | ROBINS-I | Moderate risk due to potential time-varying confounders and lack of pre-registration. | Moderate Risk |
| 67 | Kunkel genannt Bode et al. (2022) | Observational | NOS | Cross-sectional design with statistical control for some confounders. (7/9 stars) | Good Quality |
| 68 | Shen et al. (2023) | Within-subject Exp. | ROBINS-I | Moderate risk due to potential time-varying confounders and lack of pre-registration. | Moderate Risk |
| 69 | Wang et al. (2018) | Within-subject Exp. | ROBINS-I | Moderate risk due to potential time-varying confounders and lack of pre-registration. | Moderate Risk |
| 70 | Pan et al. (2021) | Within-subject Exp. | ROBINS-I | Moderate risk due to potential time-varying confounders and lack of pre-registration. | Moderate Risk |
| 71 | Zhou et al. (2018) | Observational | NOS | Case-control design (Dyslexia vs HC) with good group matching. (8/9 stars) | Good Quality |
| 72 | Lee & Kim (2020) | Within-subject Exp. | ROBINS-I | Moderate risk due to repeated-measures exposure and possible time-related confounding; objective task outcome, but blinding was not feasible. | Moderate Risk |
| 73 | Zhu et al. (2017) | Mixed design lab experiment | ROBINS-I | Moderate risk due to possible residual confounding across sessions or time of day; controlled laboratory protocol, but blinding was not feasible. | Moderate Risk |
| 74 | Ru et al. (2019) | Mixed design lab experiment | ROBINS-I | Moderate risk due to mixed within/between-subject exposure and possible residual confounding; randomised assignment and calibrated lighting were reported, but blinding was not feasible. | Moderate Risk |
| 75 | Yu & Ouyang (2024) | Within-subject Exp. | ROBINS-I | Moderate risk due to repeated testing and possible order effects; objective performance and eye-tracking outcomes were used, but blinding was not feasible. | Moderate Risk |

Supplementary Table S3 provides a visual summary of the detailed study-level risk-of-bias judgements. This plot complements the summary appraisal reported in Supplementary Table S2 and supports the interpretation of certainty constraints discussed in the main text.

**Supplementary Table S3.** Detailed Risk of Bias Judgments – Traffic Light Plot

| **Ref #** | **Author(s), year** | **Selection/Randomization** | **Confounding/Comparability** | **Intervention Bias** | **Missing Data** | **Outcome Measurement** | **Selective Reporting** |
| --- | --- | --- | --- | --- | --- | --- | --- |
| 1 | Payedar-Ardakani et al. (2024) | 🟡 | 🟡 | 🟢 | 🟢 | 🟢 | 🟡 |
| 2 | Jue & Kwon (2013) | 🟡 | 🟢 | 🟡 | 🟢 | 🟢 | 🟡 |
| 3 | Dianat et al. (2013) | 🟢 | 🔴 | 🟢 | 🟢 | 🟢 | 🟡 |
| 4 | Plass et al. (2014) | 🟡 | 🟢 | 🟡 | 🟡 | 🟢 | 🟡 |
| 5 | Rouw & Scholte (2016) | 🟢 | 🔴 | 🟢 | 🟢 | 🟢 | 🟡 |
| 6 | Daniels et al. (2020) | 🟡 | 🟡 | 🟢 | 🟢 | 🟢 | 🟡 |
| 7 | Stasenko et al. (2014) | 🟡 | 🟢 | 🟡 | 🟢 | 🟢 | 🟡 |
| 8 | Vila-López & Küster-Boluda (2019) | 🟢 | 🟢 | 🟡 | 🟢 | 🟢 | 🟡 |
| 9 | Martin et al. (2015) | 🟢 | 🟢 | 🟡 | 🟢 | 🟢 | 🟡 |
| 10 | Hagmann et al. (2016) | 🟢 | 🟡 | 🟢 | 🟢 | 🟢 | 🟢 |
| 11 | Hartstein et al. (2018) | 🟡 | 🟢 | 🟡 | 🟡 | 🟢 | 🟡 |
| 12 | Zhang et al. (2025) | 🟢 | 🔴 | 🟢 | 🟢 | 🟢 | 🟡 |
| 13 | Hu et al. (2025) | 🟢 | 🟡 | 🟢 | 🟢 | 🟢 | 🟡 |
| 14 | Heuer & Rolfs (2025) | 🟡 | 🟡 | 🟢 | 🟢 | 🟢 | 🟡 |
| 15 | Xia et al. (2016) | 🟡 | 🟢 | 🟡 | 🟡 | 🟢 | 🟡 |
| 16 | Mittelstädt et al. (2014) | 🟢 | 🟢 | 🟡 | 🟢 | 🟢 | 🟡 |
| 17 | Duan et al. (2018) | 🟡 | 🟡 | 🟢 | 🟢 | 🟢 | 🟡 |
| 18 | Xia et al. (2021) | 🟡 | 🟡 | 🟢 | 🟢 | 🟢 | 🟡 |
| 19 | Xia et al. (2023) | 🟡 | 🟡 | 🟢 | 🟢 | 🟢 | 🟡 |
| 20 | Makovski et al. (2011) | 🟡 | 🟢 | 🟢 | 🟡 | 🟢 | 🟡 |
| 21 | Årdal & Hammar (2011) | 🟢 | 🟡 | 🟢 | 🟡 | 🟢 | 🟡 |
| 22 | Diachenko et al. (2022) | 🔴 | 🔴 | 🟡 | 🟢 | 🟢 | 🟡 |
| 23 | Wang et al. (2025) | 🟡 | 🟡 | 🟢 | 🟢 | 🟢 | 🟡 |
| 24 | Zhang et al. (2022) | 🟡 | 🟡 | 🟢 | 🟢 | 🟢 | 🟡 |
| 25 | Ho et al. (2015) | 🟡 | 🟡 | 🟢 | 🟢 | 🟢 | 🟡 |
| 26 | Li et al. (2025) | 🟡 | 🟡 | 🟢 | 🟢 | 🟢 | 🟡 |
| 27 | Kress et al. (2018) | 🟡 | 🟢 | 🟡 | 🟡 | 🟢 | 🟡 |
| 28 | Lee & Chen (2025) | 🟡 | 🟡 | 🟢 | 🟢 | 🟢 | 🟡 |
| 29 | Lian et al. (2023) | 🟢 | 🟡 | 🟢 | 🟢 | 🟢 | 🟡 |
| 30 | Wang & Cho (2022) | 🟡 | 🟡 | 🟢 | 🟢 | 🟢 | 🟡 |
| 31 | Huettig et al. (2020) | 🟡 | 🟡 | 🟢 | 🟢 | 🟢 | 🟡 |
| 32 | Ooms et al. (2014) | 🟢 | 🔴 | 🟢 | 🟢 | 🟢 | 🟡 |
| 33 | Lindquist et al. (2021) | 🟡 | 🟡 | 🟢 | 🟢 | 🟢 | 🟡 |
| 34 | Min et al. (2021) | 🟡 | 🟡 | 🟢 | 🟢 | 🟢 | 🟡 |
| 35 | Takahashi et al. (2024) | 🟡 | 🟡 | 🟢 | 🟢 | 🟢 | 🟡 |
| 36 | Chai & Tang (2023) | 🟡 | 🟡 | 🟢 | 🟢 | 🟢 | 🟡 |
| 37 | Sarangi et al. (2021) | 🟡 | 🟡 | 🟢 | 🟢 | 🟢 | 🟡 |
| 38 | Chai et al. (2019) | 🟡 | 🟡 | 🟢 | 🟢 | 🟢 | 🟡 |
| 39 | Hasegawa et al. (2025) | 🟡 | 🟡 | 🟢 | 🟢 | 🟢 | 🟡 |
| 40 | M. Zhang et al. (2022) | 🟡 | 🟡 | 🟢 | 🟢 | 🟢 | 🟡 |
| 41 | Jin et al. (2022) | 🟡 | 🟡 | 🟢 | 🟢 | 🟢 | 🟡 |
| 42 | Liu et al. (2025) | 🟡 | 🟡 | 🟡 | 🟢 | 🟢 | 🟡 |
| 43 | Fang et al. (2025) | 🟡 | 🟡 | 🟢 | 🟢 | 🟢 | 🟡 |
| 44 | Lewandowska & Olejnik-Krugly (2022) | 🟡 | 🟢 | 🟡 | 🟢 | 🟢 | 🟡 |
| 45 | Wang et al. (2025) | 🟡 | 🟡 | 🟢 | 🟢 | 🟢 | 🟡 |
| 46 | Xu et al. (2025) | 🟢 | 🟡 | 🟢 | 🟢 | 🟢 | 🟡 |
| 47 | Xu et al. (2024) | 🟢 | 🟡 | 🟢 | 🟢 | 🟢 | 🟡 |
| 48 | Semin et al. (2018) | 🟢 | 🔴 | 🟢 | 🟢 | 🟢 | 🟡 |
| 49 | Matzen et al. (2021) | 🟡 | 🟡 | 🟢 | 🟢 | 🟢 | 🟡 |
| 50 | Li et al. (2024) | 🟡 | 🟢 | 🟡 | 🟢 | 🟢 | 🟡 |
| 51 | Wu et al. (2024) | 🟡 | 🟡 | 🟢 | 🟢 | 🟢 | 🟡 |
| 52 | Baudry & Gaillard (2014) | 🟢 | 🔴 | 🟢 | 🟢 | 🟢 | 🟡 |
| 53 | Kalantari et al. (2021) | 🟡 | 🟡 | 🟢 | 🟢 | 🟢 | 🟡 |
| 54 | Lim et al. (2025) | 🟢 | 🟡 | 🟢 | 🟢 | 🟢 | 🟡 |
| 55 | Bao et al. (2021) | 🟡 | 🟡 | 🟢 | 🟢 | 🟢 | 🟡 |
| 56 | Nissen et al. (2020) | 🟡 | 🟡 | 🟢 | 🟢 | 🟢 | 🟡 |
| 57 | Folgieri et al. (2013) | 🟡 | 🟡 | 🟢 | 🟢 | 🟢 | 🟡 |
| 58 | Jiang et al. (2023) | 🟡 | 🟢 | 🟡 | 🟢 | 🟢 | 🟡 |
| 59 | Hawes et al. (2012) | 🟡 | 🟢 | 🟡 | 🟢 | 🟢 | 🟡 |
| 60 | Kuzinas et al. (2016) | 🟡 | 🟡 | 🟢 | 🟢 | 🟢 | 🟡 |
| 61 | Wilms & Oberfeld (2018) | 🟡 | 🟡 | 🟢 | 🟢 | 🟢 | 🟡 |
| 62 | Jakovljević et al. (2021) | 🟢 | 🔴 | 🟢 | 🟢 | 🟢 | 🟡 |
| 63 | Laxton et al. (2023) | 🟡 | 🟡 | 🟢 | 🟢 | 🟢 | 🟡 |
| 64 | Lasauskaite & Cajochen (2017) | 🟡 | 🟢 | 🟡 | 🟡 | 🟢 | 🟡 |
| 65 | Mauderer et al. (2016) | 🟡 | 🟡 | 🟢 | 🟢 | 🟢 | 🟡 |
| 66 | Machner et al. (2020) | 🟡 | 🟡 | 🟢 | 🟢 | 🟢 | 🟡 |
| 67 | Kunkel genannt Bode et al. (2022) | 🟢 | 🟡 | 🟢 | 🟢 | 🟢 | 🟡 |
| 68 | Shen et al. (2023) | 🟡 | 🟡 | 🟢 | 🟢 | 🟢 | 🟡 |
| 69 | Wang et al. (2018) | 🟡 | 🟡 | 🟢 | 🟢 | 🟢 | 🟡 |
| 70 | Pan et al. (2021) | 🟡 | 🟡 | 🟢 | 🟢 | 🟢 | 🟡 |
| 71 | Zhou et al. (2018) | 🟢 | 🟡 | 🟢 | 🟢 | 🟢 | 🟡 |
| 72 | Lee & Kim (2020) | 🟡 | 🟡 | 🟢 | 🟢 | 🟢 | 🟡 |
| 73 | Zhu et al. (2017) | 🟡 | 🟡 | 🟢 | 🟢 | 🟡 | 🟡 |
| 74 | Ru et al. (2019) | 🟡 | 🟡 | 🟢 | 🟡 | 🟡 | 🟡 |
| 75 | Yu & Ouyang (2024) | 🟡 | 🟡 | 🟢 | 🟡 | 🟢 | 🟡 |

Legend: 🟢 Low Risk of Bias; 🟡 Moderate Risk / Some Concerns; 🔴 High Risk / Poor Quality.

**3. Appendix A. Full bibliographic details of the included studies**

Appendix A provides the full bibliographic details of the 75 studies included in the review. The numbering corresponds to the Ref # used in the deposited extraction and coding materials.

1. Payedar-Ardakani, P., et al., *Daylight illuminance levels, user preferences, and cognitive performance in office environments: Exploring an optimal illuminance range using virtual reality.* Building and Environment, 2024. **258**: p. 111638.

2. Jue, J. and S.-M. Kwon, *Does colour say something about emotions?: Laypersons’ assessments of colour drawings.* The Arts in psychotherapy, 2013. **40**(1): p. 115-119.

3. Dianat, I., et al., *Objective and subjective assessments of lighting in a hospital setting: implications for health, safety and performance.* Ergonomics, 2013. **56**(10): p. 1535-1545.

4. Plass, J.L., et al., *Emotional design in multimedia learning: Effects of shape and color on affect and learning.* Learning and Instruction, 2014. **29**: p. 128-140.

5. Rouw, R. and H.S. Scholte, *Personality and cognitive profiles of a general synesthetic trait.* Neuropsychologia, 2016. **88**: p. 35-48.

6. Daniels, E.C., A. Rodriguez, and D.L. Zabelina, *Severity of misophonia symptoms is associated with worse cognitive control when exposed to misophonia trigger sounds.* PLoS One, 2020. **15**(1): p. e0227118.

7. Stasenko, A., et al., *When concepts lose their color: A case of object-color knowledge impairment.* cortex, 2014. **58**: p. 217-238.

8. Vila-López, N. and I. Küster-Boluda, *Consumers' physiological and verbal responses towards product packages: Could these responses anticipate product choices?* Physiology & behavior, 2019. **200**: p. 166-173.

9. Martin, M.L., et al., *The experience of pain and redness in patients with moderate to severe plaque psoriasis.* Journal of Dermatological Treatment, 2015. **26**(5): p. 401-405.

10. Hagmann, C.E., et al., *Children with autism detect targets at very rapid presentation rates with similar accuracy as adults.* Journal of Autism and Developmental Disorders, 2016. **46**(5): p. 1762-1772.

11. Hartstein, L.E., M.K. LeBourgeois, and N.E. Berthier, *Light correlated color temperature and task switching performance in preschool-age children: Preliminary insights.* PLoS One, 2018. **13**(8): p. e0202973.

12. Zhang, F., et al., *Children’s physical fitness and cognitive control in China: the moderating role of family support for physical activity.* BMC Public Health, 2025. **25**(1): p. 1198.

13. Deng, H., et al., *Impaired dynamic visual attention in patients with insomnia.* Sleep Medicine, 2025: p. 106563.

14. Heuer, A. and M. Rolfs, *Predictable object motion is extrapolated to support visual working memory for surface features.* Cognition, 2025. **261**: p. 106150.

15. Xia, T., et al., *Exploring the effect of red and blue on cognitive task performances.* Frontiers in Psychology, 2016. **7**: p. 784.

16. Mittelstädt, S., A. Stoffel, and D.A. Keim. *Methods for compensating contrast effects in information visualization*. in *Computer Graphics Forum*. 2014. Wiley Online Library.

17. Duan, Y., P.A. Rhodes, and V. Cheung, *T he influence of color on impulsiveness and arousal: P art 2–C hroma.* Color Research & Application, 2018. **43**(3): p. 405-414.

18. Xia, G., et al., *Aroused and impulsive effects of colour stimuli on lateral and logical abilities.* Behavioral Sciences, 2021. **11**(2): p. 24.

19. Xia, G., et al., *The effects of colour attributes on cognitive performance and intellectual abilities in immersive virtual environments.* Computers in Human Behavior, 2023. **148**: p. 107853.

20. Makovski, T., K.M. Swallow, and Y.V. Jiang, *Attending to unrelated targets boosts short-term memory for color arrays.* Neuropsychologia, 2011. **49**(6): p. 1498-1505.

21. Årdal, G. and Å. Hammar, *Is impairment in cognitive inhibition in the acute phase of major depression irreversible? Results from a 10‐year follow‐up study.* Psychology and Psychotherapy: Theory, Research and Practice, 2011. **84**(2): p. 141-150.

22. Diachenko, I., et al., *Color education: A study on methods of influence on memory.* Heliyon, 2022. **8**(11).

23. Wang, X., et al., *Tailored information display: Effects of background colour and line spacing on visual search across different character types–An eye-tracking study.* Displays, 2025. **88**: p. 103019.

24. Zhang, L., et al., *Research on visual comfort of color environment based on the eye-tracking method in subway space.* Journal of Building Engineering, 2022. **59**: p. 105138.

25. Ho, M.-C., et al., *Numerical analysis on color preference and visual comfort from eye tracking technique.* Mathematical Problems in Engineering, 2015. **2015**(1): p. 861610.

26. Li, J., C. Wang, and M. Chen, *Effects of Driving Background Complexity and Interface Opacity on Visual Cognition in AR‐HUD Systems.* Journal of the Society for Information Display, 2025.

27. Kress, L., M. Bristle, and T. Aue, *Seeing through rose-colored glasses: How optimistic expectancies guide visual attention.* PloS one, 2018. **13**(2): p. e0193311.

28. Chen, J.-Z., *Investigating the Influence of Color on Various Perceptions Using Eye-Tracking Technology.* Frontier Computing: Volume 4: Proceedings of FC 2024, 2025. **1358**: p. 188.

29. Lian, X., et al., *The influence of picture book design on visual attention of children with autism: a pilot study.* International journal of developmental disabilities, 2023. **69**(6): p. 946-956.

30. Wang, Z.-Y. and J.Y. Cho, *Older adults’ response to color visibility in indoor residential environment using eye-tracking technology.* Sensors, 2022. **22**(22): p. 8766.

31. Huettig, F., E. Guerra, and A. Helo, *Towards understanding the task dependency of embodied language processing: The influence of colour during language-vision interactions.* Journal of Cognition, 2020. **3**(1): p. 41.

32. Ooms, K., P. De Maeyer, and V. Fack, *Study of the attentive behavior of novice and expert map users using eye tracking.* Cartography and Geographic Information Science, 2014. **41**(1): p. 37-54.

33. Lindquist, L.C., G.R. McIntire, and S.M. Haigh, *The effects of visual discomfort and chromaticity separation on neural processing during a visual task.* Vision research, 2021. **182**: p. 27-35.

34. Min, B.K., et al., *Electrophysiological Decoding of Spatial and Color Processing in Human Prefrontal Cortex.* NEUROIMAGE, 2021. **237**.

35. Takahashi, N., et al., *Temporal and spatial analysis of event-related potentials in response to color saliency differences among various color vision types.* Frontiers in Human Neuroscience, 2024. **18**: p. 1441380.

36. Chai, M.T. and T.B. Tang, *Microstates of dynamic directed connectivity networks revealing visual color influences on the brain information processing during learning.* IEEE Access, 2023. **11**: p. 14256-14273.

37. Roy, S., et al., *Brain response to color stimuli: an EEG study with nonlinear approach.* Cognitive Neurodynamics, 2021. **15**: p. 1023-1053.

38. Chai, M.T., et al., *Exploring EEG effective connectivity network in estimating influence of color on emotion and memory.* Frontiers in neuroinformatics, 2019. **13**: p. 66.

39. Hasegawa, Y., et al., *Interaction between facial expression and color in modulating ERP P3.* ENeuro, 2025. **12**(1).

40. Zhang, M., et al., *The effect of color coding and layout coding on users’ visual search on mobile map navigation icons.* Frontiers in Psychology, 2022. **13**: p. 1040533.

41. Jin, T., et al., *Combined effect of color and shape on cognitive performance.* Mathematical Problems in Engineering, 2022. **2022**(1): p. 3284313.

42. Liu, K., et al., *Effects of information framing cues and age on the comprehension of personal health records for self-care behaviors: an eye-tracking study.* Journal of the American Medical Informatics Association, 2025. **32**(7): p. 1174-1185.

43. Fang, H., et al., *The Influence of the Relationship Between Landmark Symbol Types, Annotations, and Colors on Search Performance in Mobile Maps Based on Eye Tracking.* ISPRS International Journal of Geo-Information, 2025. **14**(3): p. 129.

44. Lewandowska, A. and A. Olejnik-Krugly, *Do background colors have an impact on preferences and catch the attention of users?* Applied Sciences, 2021. **12**(1): p. 225.

45. Wang, X., et al., *How Background Colour Shapes Digital Text-Information Processing: A fNIRS-Eye Tracking Study.* International Journal of Human–Computer Interaction, 2025: p. 1-15.

46. Xu, L., et al., *Exploring the effects of background colour and gender on cognitive performance of visual attention: a multimodal approach using fNIRS and eye-tracking.* Journal of the International Colour Association, 2025. **38**: p. 30-40.

47. Xu, L., et al. *The effect of reading background colour on human cognitive performance based on multi-modal data analysis-A study of gender differences*. in *Proceedings of the Midterm Meeting of the International Colour Association*. 2024.

48. Semin, G.R., et al., *Gender is not simply a matter of black and white, or is it?* Philosophical Transactions of the Royal Society B: Biological Sciences, 2018. **373**(1752): p. 20170126.

49. Matzen, L.E., M.C. Stites, and Z.N. Gastelum, *Studying visual search without an eye tracker: An assessment of artificial foveation.* Cognitive Research: Principles and Implications, 2021. **6**(1): p. 45.

50. Li, Y., et al., *The non-visual effects of correlated color temperature on the alertness, cognition, and mood of fatigued individuals during the afternoon.* International Journal of Industrial Ergonomics, 2024. **101**: p. 103589.

51. Wu, J., et al., *Cognitive characteristics in wayfinding tasks in commercial and residential districts during daytime and nighttime: A comprehensive neuroergonomic study.* Advanced Engineering Informatics, 2024. **61**: p. 102534.

52. Baudry, S. and V. Gaillard, *Cognitive demand does not influence the responsiveness of homonymous Ia afferents pathway during postural dual task in young and elderly adults.* European journal of applied physiology, 2014. **114**(2): p. 295-303.

53. Kalantari, S., et al., *Evaluating the impacts of color, graphics, and architectural features on wayfinding in healthcare settings using EEG data and virtual response testing.* Journal of Environmental Psychology, 2022. **79**: p. 101744.

54. Lim, S., et al., *Network-based alterations in task-induced functional connectivity in bipolar disorder: An functional near-infrared spectroscopy study.* Journal of Affective Disorders, 2025: p. 119637.

55. Bao, J., et al., *Effect of lighting illuminance and colour temperature on mental workload in an office setting.* Scientific reports, 2021. **11**(1): p. 15284.

56. Nissen, A. *Psychological and Physiological Effects of Color Use on eCommerce Websites: a Neural Study Using fNIRS*. in *ICIS*. 2020.

57. Folgieri, R., C. Lucchiari, and D. Marini. *Analysis of brain activity and response to colour stimuli during learning tasks: an EEG study*. in *Color Imaging XVIII: Displaying, Processing, Hardcopy, and Applications*. 2013. SPIE.

58. Jiang, A., et al., *Short-term virtual reality simulation of the effects of space station colour and microgravity and lunar gravity on cognitive task performance and emotion.* Building and Environment, 2023. **227**: p. 109789.

59. Hawes, B.K., et al., *Effects of four workplace lighting technologies on perception, cognition and affective state.* International Journal of Industrial Ergonomics, 2012. **42**(1): p. 122-128.

60. Kuzinas, A., et al., *The effects of image hue and semantic content on viewer’s emotional Subjectives, pupil size, eye movements, and skin conductance response.* Psychology of Aesthetics, Creativity, and the Arts, 2016. **10**(3): p. 360.

61. Wilms, L. and D. Oberfeld, *Color and emotion: effects of hue, saturation, and brightness.* Psychological research, 2018. **82**(5): p. 896-914.

62. Jakovljević, T., et al., *The relation between physiological parameters and colour modifications in text background and overlay during reading in children with and without dyslexia.* Brain sciences, 2021. **11**(5): p. 539.

63. Laxton, V., et al., *Standardised colour-coded compartmentalised syringe trays improve anaesthetic medication visual search and mitigate cognitive load.* British Journal of Anaesthesia, 2023. **130**(3): p. 343-350.

64. Lasauskaite, R. and C. Cajochen, *Influence of lighting color temperature on effort-related cardiac response.* Biological psychology, 2018. **132**: p. 64-70.

65. Mauderer, M., D.R. Flatla, and M.A. Nacenta. *Gaze-contingent manipulation of color perception*. in *Proceedings of the 2016 CHI Conference on Human Factors in Computing Systems*. 2016.

66. Machner, B., et al., *Unbalancing the attentional priority map via gaze-contingent displays induces neglect-like visual exploration.* Frontiers in Human Neuroscience, 2020. **14**: p. 41.

67. Kunkel genannt Bode, L., et al., *Gaze-contingent display technology can help to reduce the ipsilesional attention bias in hemispatial neglect following stroke.* Journal of NeuroEngineering and Rehabilitation, 2022. **19**(1): p. 125.

68. Shen, H., et al., *Evaluating the efficacy of using a novel gaze-based attentive user interface to extend ADHD children’s attention span.* International journal of human-computer studies, 2023. **169**: p. 102927.

69. Wang, Q., et al., *The applicability of eye‐controlled highlighting to the field of visual searching.* Australian journal of psychology, 2018. **70**(3): p. 294-301.

70. Pan, Y., et al., *Using eye-controlled highlighting techniques to support both serial and parallel processing in visual search.* Applied Ergonomics, 2021. **97**: p. 103522.

71. Zhou, W., et al., *Word segmentation by alternating colors facilitates eye guidance in Chinese reading.* Memory & cognition, 2018. **46**(5): p. 729-740.

72. Lee, C.W. and J.H. Kim, *Effect of LED lighting illuminance and correlated color temperature on working memory.* International Journal of Optics, 2020. **2020**(1): p. 3250364.

73. Zhu, Y., et al., *Effects of illuminance and correlated color temperature on daytime cognitive performance, subjective mood, and alertness in healthy adults.* Environment and Behavior, 2019. **51**(2): p. 199-230.

74. Ru, T., et al., *Non-image forming effects of illuminance and correlated color temperature of office light on alertness, mood, and performance across cognitive domains.* Building and Environment, 2019. **149**: p. 253-263.

75. Yu, N. and Z. Ouyang, *Effects of background colour, polarity, and saturation on digital icon status recognition and visual search performance.* Ergonomics, 2024. **67**(3): p. 433-445.
